# Triggering Receptor Expressed on Myeloid Cells-1 Agonist Regulates Intestinal Inflammation via Cd177^+^ Neutrophils

**DOI:** 10.3389/fimmu.2021.650864

**Published:** 2021-03-09

**Authors:** Dong Hyuk Seo, Xiumei Che, Soochan Kim, Da Hye Kim, Hyun Woo Ma, Jae Hyeon Kim, Tae Il Kim, Won Ho Kim, Seung Won Kim, Jae Hee Cheon

**Affiliations:** ^1^Department of Internal Medicine and Institute of Gastroenterology, Yonsei University College of Medicine, Seoul, South Korea; ^2^Brain Korea 21 PLUS Project for Medical Science, Yonsei University College of Medicine, Seoul, South Korea; ^3^Severance Biomedical Science Institute, Yonsei University College of Medicine, Seoul, South Korea

**Keywords:** CD177, inflammatory bowel disease, neutrophil, macrophage, triggering receptor expressed on myeloid cell

## Abstract

Triggering receptor expressed on myeloid cell-1 (TREM-1) signaling is expressed on neutrophils and monocytes that is necessary for the successful antimicrobial response and resolution of inflammation in the gut. In this study, we determined the effect of an anti-TREM-1 agonistic antibody (α-TREM-1) on colitis and identify its underlying mechanism of action. Administration of α-TREM-1 alleviated colitis in mice and resolved dysbiosis, which required TLR4/Myd88 signaling. α-TREM-1 increased the production of neutrophil extracellular traps and interleukin-22 by CD177^+^ neutrophils, which led to pathogen clearance and protection of the intestinal barrier. TREM-1 activation using an α-TREM-1 antibody protects against colitis by rebalancing the microbiota and protecting the epithelium against the immune response as well as modulates the function of neutrophils and macrophages. These results highlight the importance of the TREM-1 pathway in intestinal homeostasis and suggest that α-TREM-1 treatment may be an effective therapeutic strategy for inflammatory bowel disease.

## Introduction

The gut is constantly exposed to microbes. Unresolved pathogen clearance due to aberrant immune responses and compromised mucosal healing perpetuate inflammation and tissue injury in the gut. One factor determining the balance between bacterial clearance and tissue damage is the timely induction of anti-inflammatory and mucosal healing molecules. Inflammatory bowel diseases (IBDs), including Crohn's disease (CD) and ulcerative colitis (UC), are chronic inflammatory disorders related to dysregulated immune responses, genetic susceptibility, and environmental factors (1, 2). Recent studies have shown the importance of aberrant innate immune responses to microbes in IBD pathogenesis (3). Myeloid cells, such as neutrophils, monocytes/macrophages, and dendritic cells, primarily mediate this innate response (4). Excessive inflammation due to unresolved infection, however, leads to prolonged inflammation and tissue damage.

Triggering receptor expressed on myeloid cells-1 (TREM-1) is expressed mainly primarily on myeloid cells, such as including neutrophils, monocytes, and tissue macrophages (5), and is dramatically induced on neutrophils and monocytes in response to microbes, playing a critical role in modulating infection-induced inflammation (6). TREM-1 downstream signaling is linked with the phosphorylated DNAX activation protein 12 (DAP12), phosphatidylinositol-3 kinase (PI3K), and extracellular-signal-regulated kinase (ERK) in order to amplify Toll-like receptors (TLRs). TREM-1 amplifies TLR signaling, an important link between microbial populations and inflammation (6). Although the exact ligand for TREM-1 is unknown, cross-linking with an agonist antibody (α-TREM-1) induces TREM-1-dependent responses, including increased cytokine production, bactericidal activity, and phagocytosis in monocytes, and promotion of degranulation and antimicrobial function in neutrophils (6). Modulation of the TREM-1 pathway has been shown to alter outcomes in several animal models of inflammation (7, 8). Moreover, in a preclinical trial of anti-TREM-1 therapy, the secretion of several proinflammatory cytokines was suppressed in the inflamed intestinal tissues of IBD patients (9). However, it has also been reported that a TREM-1-antagonizing peptide attenuates colitis in mice (10). Thus, the exact role of TREM-1 in driving chronic inflammation in IBD is poorly understood.

We aimed to determine the effect of α-TREM-1 on intestinal inflammation and explored its underlying mechanism of action. We showed that TREM-1 is indispensable for the innate immune response and barrier function in colitis. Furthermore, we showed that α-TREM-1 induced CD177^+^ neutrophils and promoted wound healing through interactions with macrophages and intestinal epithelial cells (IEC).

## Materials and Methods

### Colitis and Animal Models

**Figure 1:**
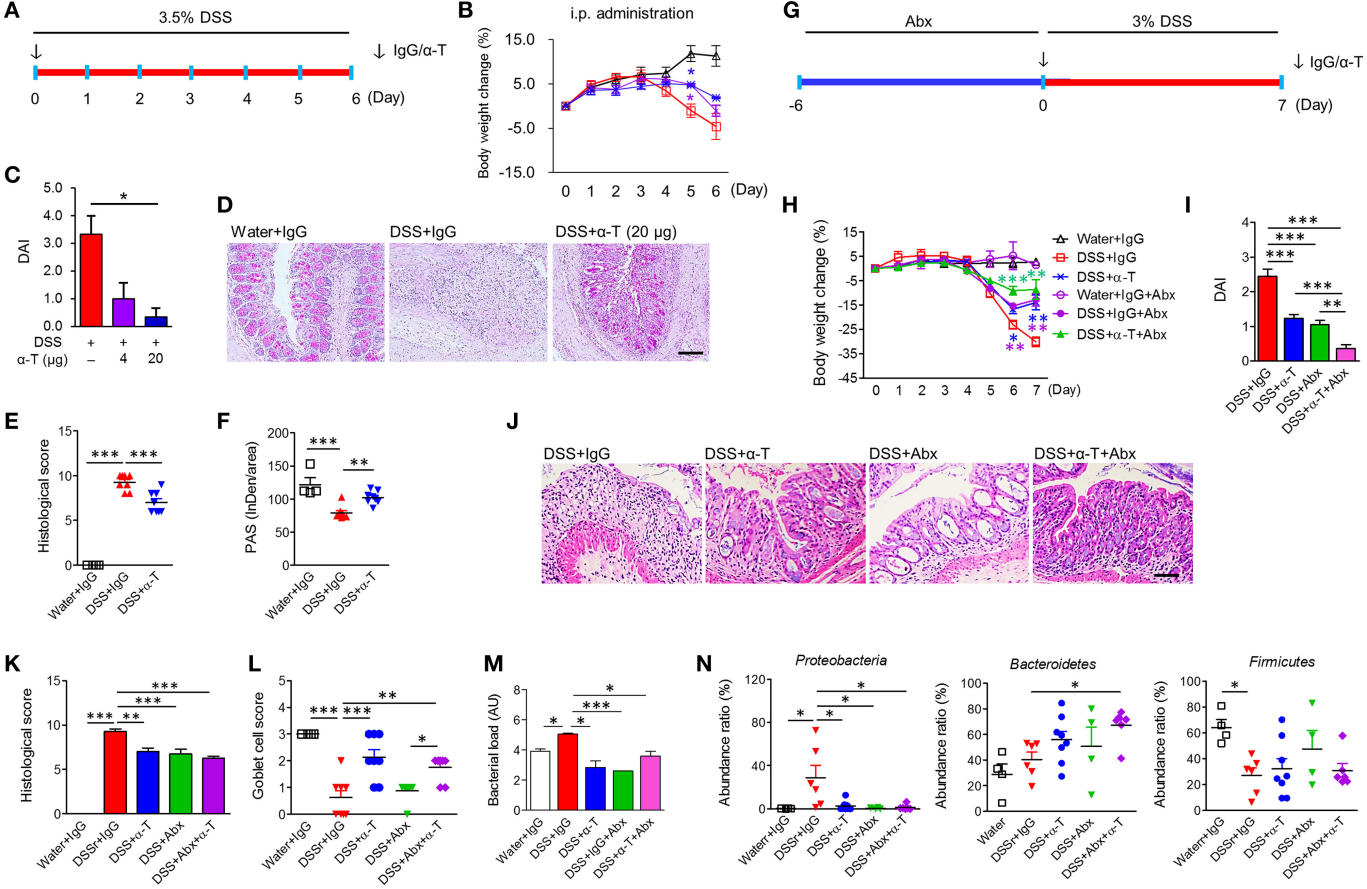
α-TREM-1 prevents colitis and modulates the microbiota in mice. **(A**–**F)** Systemic administration of α-TREM-1 in DSS-treated mice. DSS was supplied in drinking water and IgG or α-TREM-1 was intraperitoneally injected (4 or 20 μg/mouse) into BALB/c mice. **(G**–**N)** The antibiotic (Abx) cocktail was supplied in drinking water 6 days before DSS administration (day 0) and IgG or α-TREM-1 (arrow: 20 μg/mouse) was intraperitoneally injected into BALB/c mice on day 0 (*n* = 8/groups). **(A**,**G)** Experimental design. **(B**,**H)** Body weight change. **(C**,**I)** Disease activity index. **(D**,**J)** Representative sections of periodic acid-Schiff stain. Scale bar, 100 μm. **(E**,**K)** Histological score. **(F)** PAS stain intensity. **(L)** Goblet cell score. **(M)** Bacterial load. Bacterial load in colon was assessed by 16S rRNA gene amplification. **(N)** Microbiota profiles (phylum level) in colonic tissues. Data are expressed as means ± S.E.M. (*n* = 4–8/groups). Statistical significance was assessed using one-way ANOVA followed by Tukey post-test. *P < 0.05, **P < 0.01, ***P < 0.005 (or vs. DSS+IgG). α-T, treated with α-TREM-1; IgG, treated with control antibody; Water, supplied with normal drinking water.

**Figure 2:**
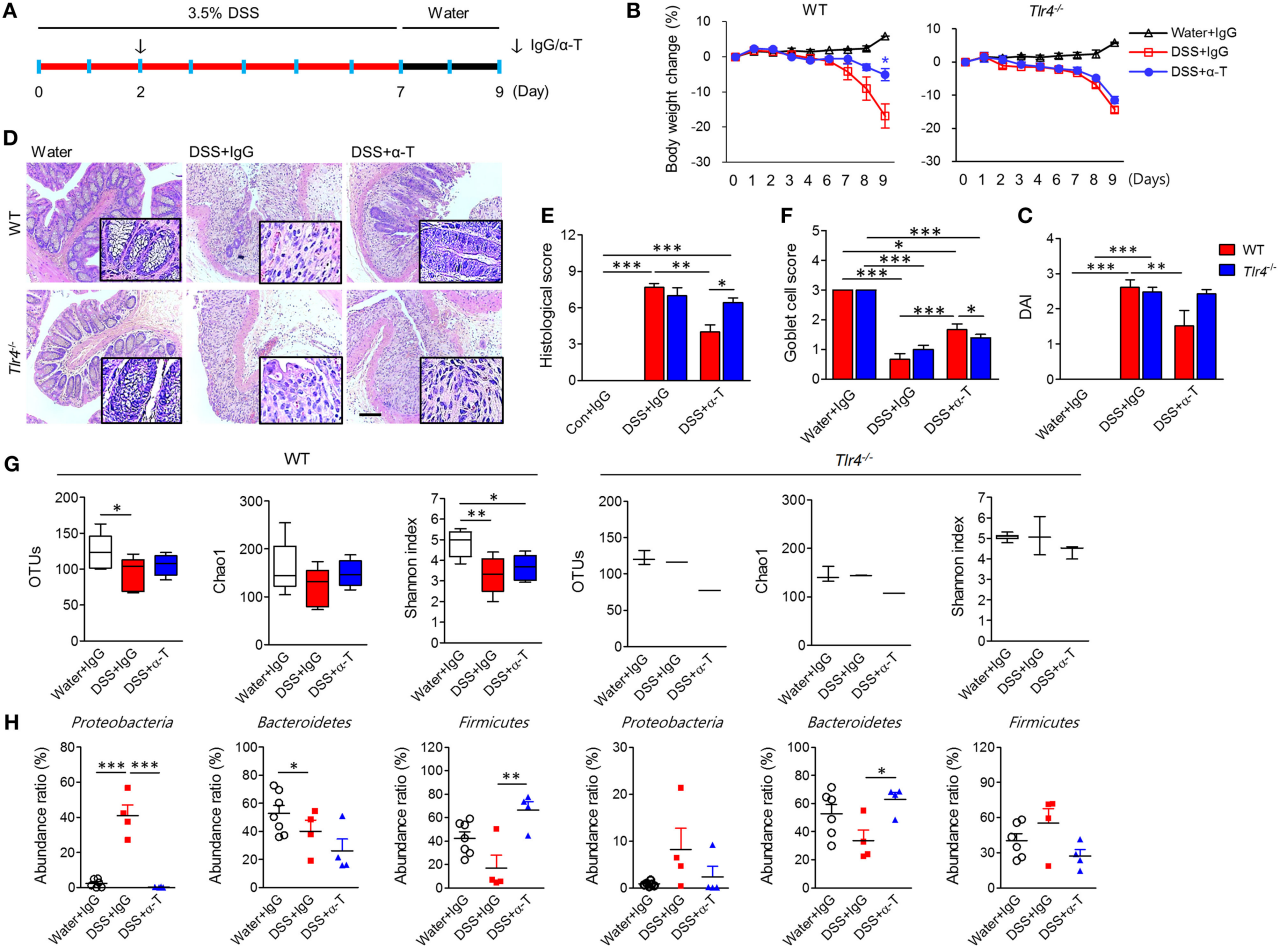
TLR4 signaling is required for the anticolitic effects of α-TREM-1. Wild-type (WT) and *Tlr4*-knockout (*Tlr4*^−/−^) BALB/c mice were subjected to a colitis and healing model with 3.5% DSS treatment for 7 days and normal drinking water for 2 days (*n* = 7/groups). The arrow indicates the point at which IgG- or α-TREM-1 (α-T) was administered (20 μg/mouse). **(A)** Experimental design. **(B)** Body weight change. **(C)** Disease activity index. **(D)** Representative sections of PAS stain. Scale bar, 100 μm. **(E)** Histological score. **(F)** Goblet cell score. **(G)** Total number of bacterial OTUs (left), richness predicted by the Chao1 index (middle), and diversity by the Shannon index (right) in the colon. **(H)** Microbiota profiles in the colon at the phylum level. Statistical significance was assessed using one-way ANOVA followed by Tukey post-test. *P < 0.05, **P < 0.01, ***P < 0.005. PBS administered phosphate-buffered saline; Water, supplied with normal drinking water.

TLR4-deficient BALB/c and Myd88-deficient C57BL/6 mice were provided by the Korea Research Institute of Bioscience and Biotechnology (Daejeon, South Korea). dextran sodium sulfate (DSS) (MP Biomedicals, Solon, OH, USA) or 2,4,6-trinitrobenzene sulfonic acid (TNBS) (Thermo Fisher Scientific, Waltham, MA, USA) were used to induce colitis and analysis was performed as previously described (11). At the time of DSS or TNBS treatment (day 0), we administrated an isotype control (IgG; R&D Systems, Minneapolis, MN, USA); three different α-TREM-1 (4 or 20 μg/mouse based on a previous study) (7); MAB1187 (R&D Systems) for experiments in C57BL/6 mice (Figure 1); AF1187 (R&D Systems) for all experiments, except those in Figure 2; or sc-19312 (Santa Cruz Biotechnology, Dallas, TX, USA) for the indicated experiments in BALB/c mice in Figure 2. All experiments using animals were approved by the Institutional Animal Care and Use Committee of Yonsei University Severance Hospital, Seoul, Korea (Approval No: 2014-0299).

The detailed methods for disease activity index (DAI) evaluation, histological analysis and immunohistochemistry, depletion or transfer experiments of microbiota, and metagenome analysis of microbiota are described in Supporting Information.

### Cell Culture, Treatment, and Transfection

Cells were maintained at 37°C in RPMI1640 supplemented with 10% heat-inactivated fetal bovine serum (FBS; Life Technologies, Gaithersburg, MD, USA) and 1% antibiotics in a humidified atmosphere of 5% CO_2_. RAW264.7 macrophage cells (Korean Cell Line Bank, Seoul, Korea), THP-1 cells, and HL-60 cells (ATCC, Manassas, VA, USA) were used. Cells were stimulated with IgG or α-TREM-1 at 0.4–0.8 μg/mL, with or without TLR ligands, including LPS (Sigma-Aldrich, St Louis, MO, USA), flagellin (FLA-ST; InvivoGen, San Diego, CA, USA), Pam3CSK4 (PAM3; InvivoGen), peptidoglycan (PGN-BS; InvivoGen), muramyl dipeptide (MDP; InvivoGen), and *Salmonella enterica* serovar *typhimurium* expressing green fluorescent protein (GFP; ATCC14028GFP). *S. typhimurium* expressing GFP was inoculated into 10 mL of Luria-Bertani broth at 37°C, shaken at 250 rpm overnight, and then sub cultured into 50 mL of LB broth, until mid-logarithmic growth was reached (OD_600_: 0.5) as previously described (12). *S. typhimurium* was then diluted in antibiotic-free medium.

Knockdown of the *Trem1* gene was achieved through a 12-h transfection of siRNA or non-targeting control siRNA (40 μM; AccuTarget, Bioneer, Daejeon, South Korea) into RAW264.7 cells using Lipofectamine 2000 (Life Technologies). To assess the inflammatory response, treatment was performed 24 h after transfection. Transfection experiments were performed in duplicate on three independent occasions and the results were averaged.

The detailed methods of bone marrow–derived neutrophil and macrophage preparation are described in the Supporting Information.

### Phagolysosomal Acidification, Autophagy, and Neutrophil Extracellular Trap Assay

Macrophages and neutrophils were cultured on poly-L-lysine-coated confocal dishes and incubated with LPS (200 ng/mL) or α-TREM-1 (0.8 μg/mL) for 2 or 3 h, respectively. For the phagolysosomal acidification assay, live cells were treated with 100 nM LysoTracker Red DND-99 (Thermo Fisher Scientific) for 30 min, washed with PBS, and stained with Hoechst 33342 (8 nmol/L, Thermo Fisher Scientific). For autophagosome evaluation, cells were permeabilized with 0.1% Triton X-100 for 10 min, washed with PBS, and incubated with an anti-LC3B antibody (1:2,000; Abcam, Cambridge, UK) overnight. An Alexa488-conjugated rabbit anti-mouse secondary antibody (1:500, Thermo Fisher Scientific) was then added for 30 min, after which cells were fixed in 4% paraformaldehyde and stained with DAPI or Hoechst 33342. For the neutrophil extracellular trap assay, neutrophils were treated with 5 μM SYTOX orange (Thermo Fisher Scientific) for 30 min. All cells were visualized using a fluorescence microscopy (Olympus BX41) or Carl Zeiss LSM 700 laser-scanning microscope (Oberkochen, Germany). At least 100 cells were counted in 10 high-powered fields.

The detailed methods of culture, knockdown, transfection, neutrophil isolation, flow cytometric analysis, reactive oxygen species measurement, RT-PCR, Western blotting, wound healing assay, and immunostaining are described in Supporting Information. Supplementary Table 1 summarizes the patient characteristics. qPCR primers are listed in Supplementary Table 2. This study was approved by the Institutional Review Board of Severance Hospital, Yonsei University (approval number 4-2012-0302). All patients and controls provided written informed consent and all methods were performed in accordance with the relevant guidelines and regulations.

### Statistical Analysis

Prism 5.0 software (GraphPad Inc., San Diego, CA, USA) was used for statistical analyses. A two-tailed Student's *t*-test was used to compare two datasets and analysis of variance (ANOVA) was used for multiple comparisons. Significance was accepted at *P* < 0.05. Results are expressed as mean ± S.E.M.

## Results

### Intrarectal and Intraperitoneal Administration of α-TREM-1 Protects Mice From Colitis

We examined the effect of TREM-1 agonism by direct intrarectal administration of α-TREM-1 agonist at the time of TNBS treatment (day 0) into C57BL/6 mice (Supplementary Figure 1A). Unexpectedly, we found that α-TREM-1 induced body weight recovery, lowered DAI values, and attenuated colon length shortening in a dose-dependent manner (Supplementary Figures 1B–D). α-TREM-1 alleviated histopathological changes (Supplementary Figures 1E–G), suggesting that α-TREM-1 can directly impact mucosal immunity through rectal administration. The TNBS-induced colitis model that haptenates to the host immune system in the intestine has been considered as a Th1-mediated CD-like colitis model, and the DSS-induced colitis model that causes massive colonic barrier loss has been considered a model of UC-like disease (13, 14). To test whether α-TREM-1 had systemic effects, we intraperitoneally administered α-TREM-1 at the time of DSS treatment (day 0) to mice (Figure 1A). Like intrarectal administration, α-TREM-1 dose-dependently attenuated colitis (Figures 1B,C, Supplementary Figure 2A). Histological evaluation of colons from α-TREM-1-treated mice revealed a markedly decreased inflammation score (Figures 1D–F) and showed near complete goblet cell restoration (Figures 1D,F). To ensure these results were TREM-1 specific, we used three different α-TREM-1 agonists with different epitopes (described in the Methods section). The anti-colitic effects of α-TREM-1 were not affected by the type of antibody. These results confirmed that α-TREM-1 specifically activated TREM-1 signaling and induced anticolitic effects through intrarectal and intraperitoneal administration, suggesting the involvement of systemic modulators, such as neutrophils and macrophages, in addition to mucosal immunity.

### α-TREM-1 Promotes Bacterial Clearance and Modulates Microbiota in Colitis

To further examine the involvement of bacterial modulation of the anticolitic effects of α-TREM-1, we depleted endogenous intestinal bacteria with broad-spectrum antibiotic (Abx) treatment (Supplementary Figure 3A) and intraperitoneally administered IgG or α-TREM-1 at the time of DSS treatment (Figure 2G). Here, we used BALB/c mice to investigate the effects of the mouse genetic background. Like α-TREM-1 treatment, Abx treatment alleviated colitis (Figures 1H–L, Supplementary Figure 3B) in DSS-treated mice. Notably, α-TREM-1 yielded stronger anticolitic effects when co-administered with antibiotics. Next, we investigated bacterial burden in colonic tissues. Bacterial load was significantly reduced in the DSS+α-TREM-1, DSS+Abx+α-TREM-1, and DSS+Abx groups compared to the DSS+IgG group (Figure 1M). Correspondingly, we found that α-TREM-1 also reduced colonic bacterial load in mice with TNBS-induced colitis (Supplementary Figure 1H).

We evaluated changes in fecal and colonic microbiota using 16S pyrosequencing. Feces from the Abx-treated group showed compositional shifts to *Bacteroidetes* (Supplementary Figures 3C,D), which is supposed to be due to Abx treatment. Colon samples from α-TREM-1- and Abx-treated mice revealed a different taxa composition compared to those of IgG-treated mice, with a drastic decrease in *Proteobacteria* and an increase in *Firmicutes* in DSS-treated mice (Figure 1N, Supplementary Figure 3E). Similar results were found in the TNBS-treated mice (Supplementary Figures 1I–L), although *Bacteroidetes* displayed slightly different profiles between the DSS and TNBS models. These results indicate that α-TREM-1-mediated attenuation of colitis was associated with restricted dissemination of pathogenic bacteria and a change toward non-pathogenic compositions in the early stage of inflammation.

### TLR4 Signaling Is Required for Anticolitic Effects of α-TREM-1

Because the interaction between TREM-1 and TLR4 is crucial for antimicrobial and anti-inflammatory functions, we investigated the therapeutic effects of α-TREM administration in *Tlr4*- and *Myd88*-knockout (KO) BALB/c mice through intraperitoneal administration of α-TREM-1 2 days after DSS treatment (Figure 2A, Supplementary Figure 5A). As seen in C57BL/6 mice, α-TREM-1 markedly attenuated colitis in wild type (WT) BALB/c mice. However, α-TREM-1 treatment did not alleviate colitis in *Tlr4*- and *Myd88*-KO mice (Figures 2B,C, Supplementary Figures 4, 5). It was also unable to restore goblet cells or improve histopathology in *Tlr4*-KO mice (Figures 2D,E) and *Myd88*-KO mice (Supplementary Figure 5E). Additionally, α-TREM-1-treated WT mice showed an increase in microbiota diversity (Figure 2G, Supplementary Figure 4C). This therapeutic model also showed a shift to *Firmicutes* in the colonic tissue of α-TREM-1-treated WT mice, but significant suppression of pathogenic *Proteobacteria* in DSS-treated mice. This shift was absent in *Tlr4*- or *Myd88*-KO mice (Figure 2H, Supplementary Figures 4D, 5F). α-TREM-1 treatment drastically increased expression levels of genes related to bacterial clearance, such as *Inos* and *Il1b*, in the affected colon of WT mice compared to untreated mice. In addition, α-TREM-1 treatment resulted in a trend toward increased *Il22* expression (Supplementary Figure 4E). On the other hand, α-TREM-1 treatment did not affect *Inos, Il1b*, or *Il22* expression in *Tlr4*-KO mice. These results suggest that TLR4 signaling is associated with the anticolitic effects of α-TREM-1 through the control of gut microbiota.

**Figure 3:**
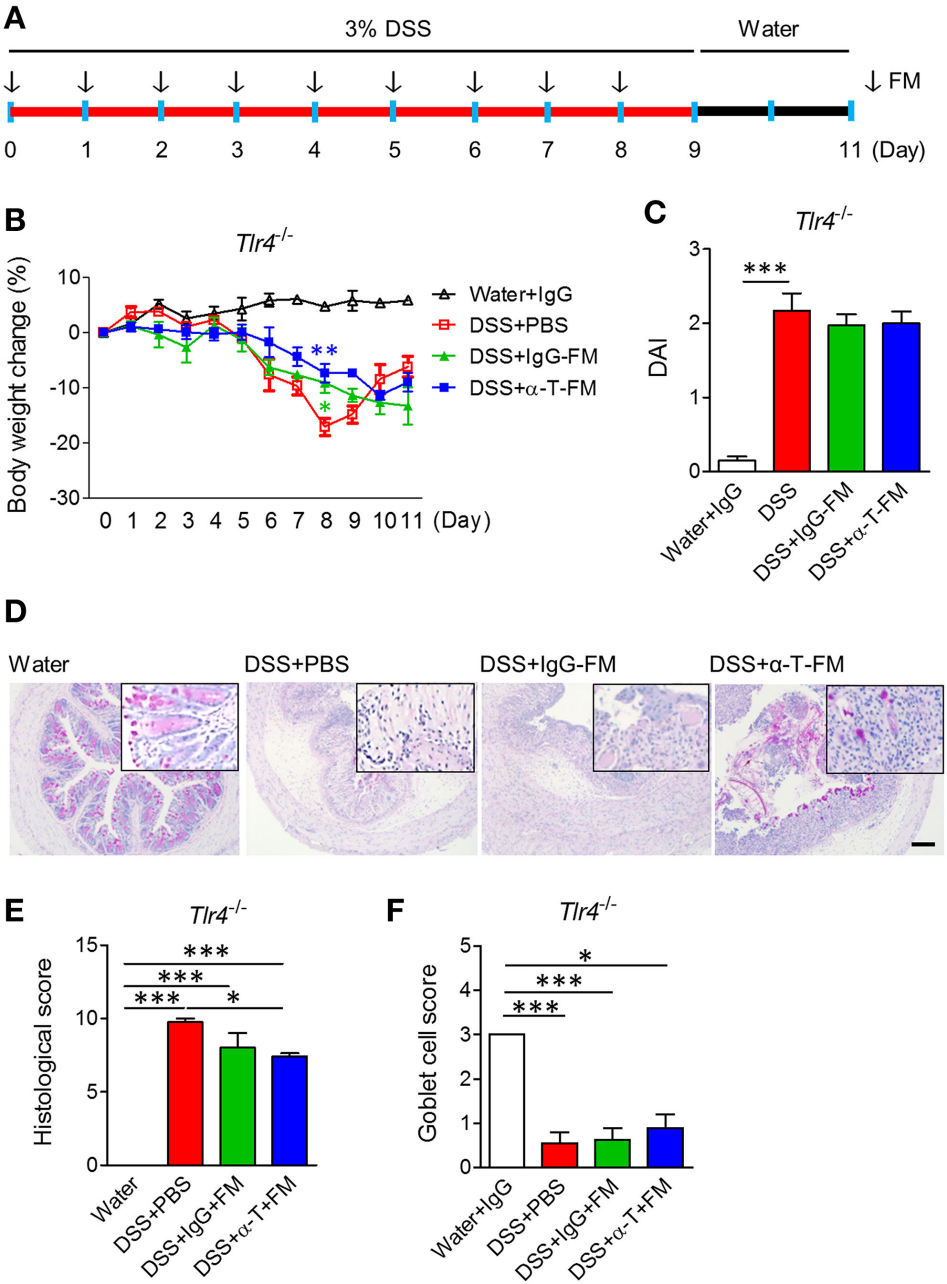
Fecal microbiota transplantation. WT mice were intraperitoneally treated with IgG- and α-TREM-1 6 times weekly and mouse feces were collected daily. *Tlr4*-knockout mice were orally administered fecal microbiota (FM) from the treated mice daily for 8 days after 3% DSS treatment, and DSS was changed with drinking water for 2 days until the endpoint of the experiment (*n* = 5/groups). The arrow indicates the point at which FM was administered. **(A)** Experimental design. **(B)** Body weight change. **(C)** Disease activity index. **(D)** Representative sections of PAS stain. Scale bar, 100 μm. **(E)** Histological score. **(F)** Goblet cell score. Statistical significance was assessed using one-way ANOVA followed by Tukey post-test. *P < 0.05, **P < 0.01, ***P < 0.005. PBS administered phosphate-buffered saline; Water, supplied with normal drinking water.

To assess whether the nullified anticolitic effects of α-TREM-1 in *Tlr4*-KO mice were due to gut microbiota changes, we cohoused *Tlr4*-KO mice with WT mice that were intraperitoneally treated with IgG- and α-TREM-1 6 times weekly to facilitate the exchange of microbiota (Supplementary Figure 6A). Body weight recovered until day 8 of DSS-treatment in α-TREM-1-treated mice, but this effect disappeared at the end of the observation period (Supplementary Figure 6B). Cohousing did not improve DAI values, colon length, or histopathology (Supplementary Figures 6C–G), and there was no induction of IL-22-producing neutrophils or M2 macrophages in α-TREM-1-treated mice (Supplementary Figures 6H,I). Next, we performed daily oral fecal microbiota transplantation (FMT) to DSS-treated mice for 9 days using feces obtained from cohousing experiments (Figure 3A). FMT from IgG- and α-TREM-1-treated mice alleviated body weight loss until day 8, but *Tlr4*-KO mice rapidly became debilitated when FMT ceased (Figure 3B). In addition, DAI values and colon length were similar between all DSS-treated groups (Figure 3C, Supplementary Figure 7A), despite a slight restoration of goblet cells and histology in the colons of α-TREM-1-treated *Tlr4*-KO mice (Figures 3D–F). FMT of WT mice treated with α-TREM-1 to DSS-treated *Myd88*-KO mice yielded similar observations to that of *Tlr4*-KO mice (Supplementary Figures 7B–H). Overall, fecal microbiota appeared to have a temporary effect on α-TREM-1, suggesting that other basic host elements are critical in the anti-colitic effect of α-TREM-1.

### Anticolitic Effects of α-TREM-1 Are Mediated by Neutrophils and Macrophages

**Figure 4:**
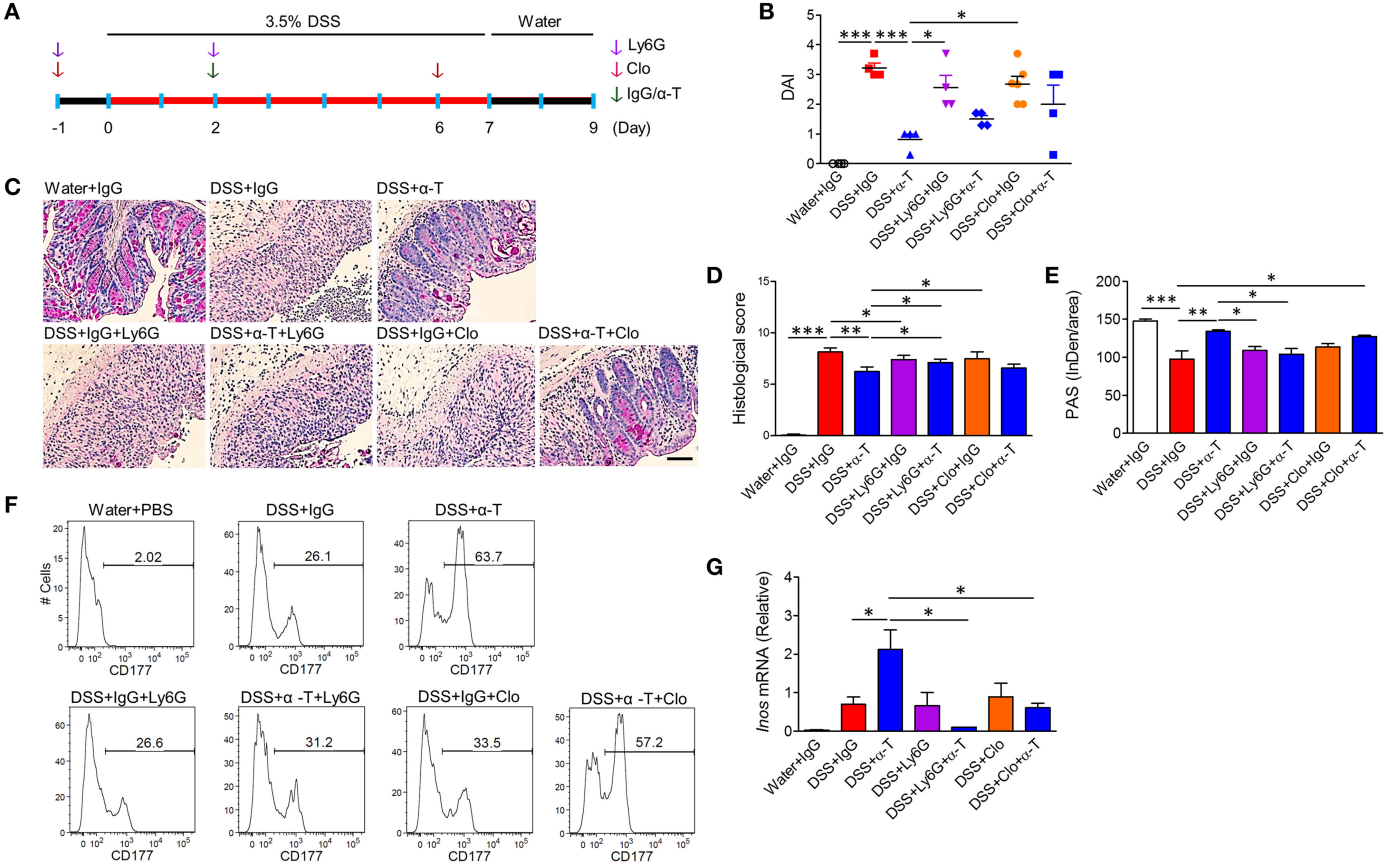
Neutrophils and macrophages mediate α-TREM-1-induced anti-colitic effects. BALB/c mice were intraperitoneally given an anti-Gr1 antibody (Ly6G) twice to deplete neutrophils or clodronate (Clo) twice to deplete macrophages. Both these groups were then injected once with IgG or α-TREM-1 (20 μg/mouse) at day 2. After DSS treatment for 8 days, DSS was exchanged for drinking water for 2 days (*n* = 4–6/groups). **(A)** Experimental design. **(B)** Disease activity index. **(C)** Representative sections of a periodic acid-Schiff stain. Scale bar, 100 μm. **(D)** Histological score. **(E)** Densitometry analysis of periodic acid-Schiff stain. **(F)** Flow cytometric analysis of macrophages and CD177^+^ neutrophils in lamina propria mononuclear cells. **(G)** qRT-PCR analysis of Inos expression profiles in colons. Each data represents the mean of duplicate real-time RT-PCR (*n* = 3–4). Data are expressed as means ± S.E.M. Statistical significance was assessed using one-way ANOVA followed by Tukey post-test **(B**,**G)** or Student *t*-test **(D**,**E)**. *P < 0.05, **P < 0.01, ***P < 0.005. α-T, treated with α-TREM-1; IgG, treated with control antibody; Water, supplied with normal drinking water.

TREM-1 is primarily expressed in cells of myeloid origin, such as neutrophils and macrophages (4). Therefore, we depleted neutrophils or macrophages with Ly6G antibody or clodronate liposome treatment, respectively, and assessed whether α-TREM-1-treated mice were still susceptible to DSS-induced colitis (Figure 4A, Supplementary Figure 8D). Colitis worsened in Ly6G- and clodronate-treated mice (Figures 4B–D). Moreover, histopathological scores and goblet cells were significantly different between α-TREM-1-treated mice treated with vehicle and those treated with Ly6G (Figures 4C–E), demonstrating that neutrophils and macrophages were required for the protective effect of α-TREM-1. However, clodronate treatment was not sufficient to block α-TREM-1 effects and induce IL-22-producing CD177^+^ neutrophils (Figure 4F). Of note, the increase in *iNos* expression induced by α-TREM-1 was abolished by Ly6G treatment (Figure 4G). This suggests that the presence of macrophages was insufficient to mediate the anticolitic effects of α-TREM-1, but the presence of neutrophils was critical.

### α-TREM-1 Promotes Bacterial Clearance by Modulating Neutrophil and Macrophage Function

**Figure 5:**
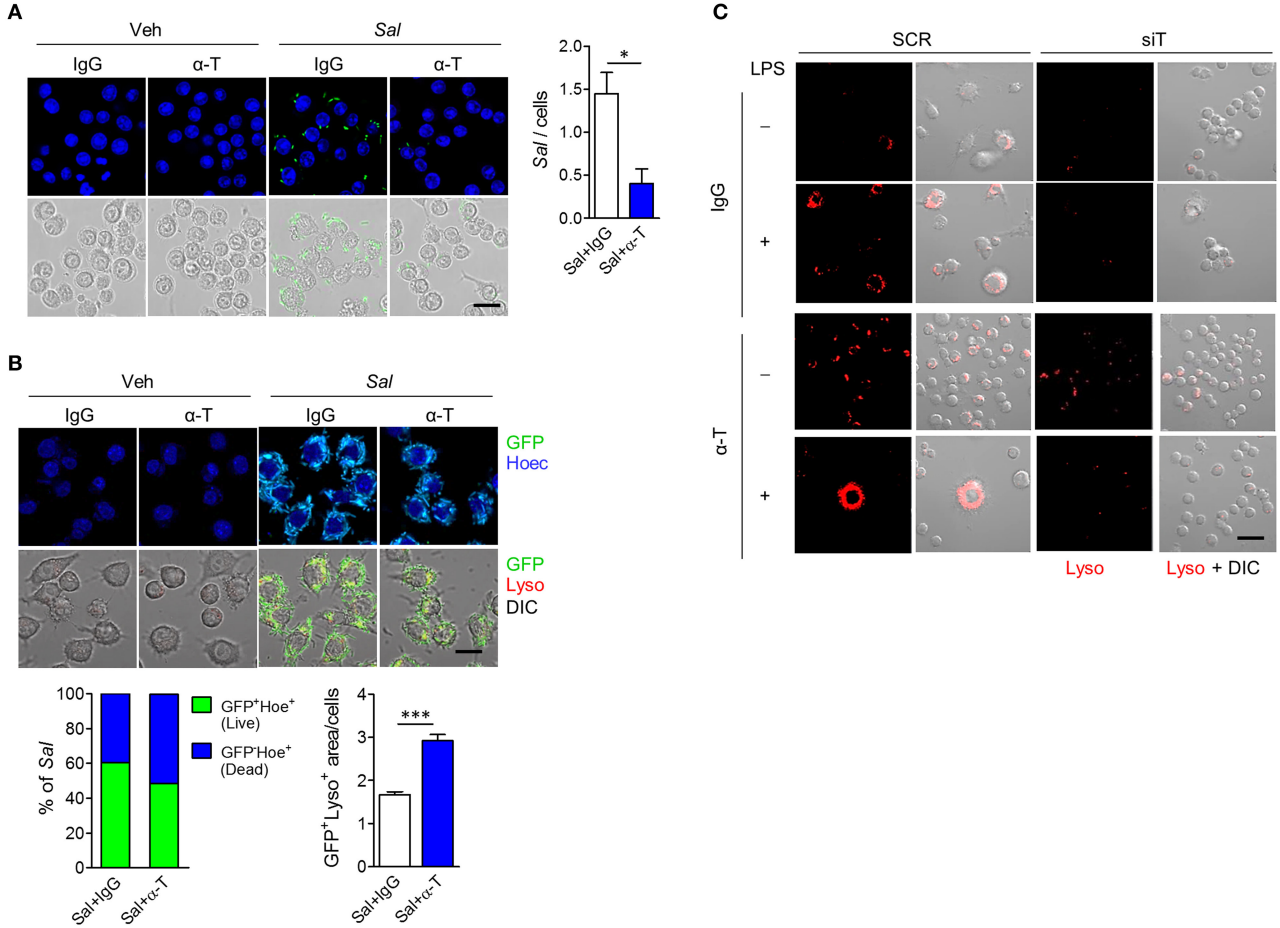
α-TREM-1 promotes bacterial clearance through increased macrophage autophagy. **(A**,**B)** RAW264.7 cells were pre-treated with IgG or α-TREM-1 and infected with *S. typhimurium* expressing green-fluorescent protein (**A**, multiplicity of infection MOI = 10; B, MOI = 100) for 1 h. **(A)** Representative images of *S. typhimurium*-GFP-infected macrophages and quantification of the total number of bacteria per macrophage (right). Scale bar, 20 μm. **(B)** Representative images of lysotracker (Lyso)-stained macrophages and the percentage of *S. typhimurium*-GFP in autophagic degradation (GFP co-localization with lysosomes). GFP^+^Lyso^+^ area was calculated by subtracting pure green GFP signal from total GFP signal. Scale bar, 20 μm. Hoechst (Hoec) staining of nuclei. **(C)** Representative images of lysotracker-stained macrophages. Scale bar, 40 μm. RAW264.7 cells transfected with scrambled (SCR) or *TREM1*-specific (siT) siRNA were stimulated with LPS for 4 h after pre-treatment with α-TREM-1. Data are expressed as means ± S.E.M. of at least three independent experiments. Statistical significance was assessed using Student *t*-test. *P < 0.05, ***P < 0.005. α-T, treated with α-TREM-1; DIC, differential interference contrast; IgG, treated with control antibody; *Sal*, infected with *S. typhimurium-*GFP; Veh, treated with vehicle.

Since IBD is associated with many genetic variants that affect bacterial clearance, such as NOD2, and autophagy, such as ATG16L1 (15), we assumed that increased bacterial clearance after α-TREM-1 treatment resulted from increased autophagy. To assess the effect of α-TREM-1 on bacterial clearance in macrophages, we infected RAW264.7 cells with live GFP-expressing *S. typhimurium*, an invasive intestinal pathogen. Intracellular bacteria were detected as GFP (live) and Hoechst (dead) signals as previously described (15). α-TREM-1 treatment significantly increased the percentage of dead bacteria (Figures 5A,B, Supplementary Figures 9A–C). Since *S. typhimurium* lipopolysaccharide (LPS), a TLR4 ligand, induces autophagy in RAW264.7 cells, we stained lysosomes and LC3B using a lysotracker probe (or anti-LAMP-1) and an anti-LC3B antibody, respectively. We observed significantly increased GFP signal localized to lysosomes in α-TREM-1-treated cells (Figure 5B, Supplementary Figures 9B,C), indicating increased fusion of *S. typhimurium*-containing phagosomes with lysosomes and confirming decreased bacterial survival after α-TREM-1 treatment. We found an increase in LC3-II level and endogenous processing of lysosomes in α-TREM-1-treated cells using immunostaining and western blotting, respectively (Supplementary Figures 9D–F). Short interfering RNA (siRNA) was used to knock down *Trem1*. α-TREM-1-treated RAW264.7 cells showed increased lysotracker levels, but not in *Trem1-*knockdown cells (Figure 5C). Collectively, these results show that α-TREM-1 further activates phagocytosis by macrophages.

### Induction of CD177^+^ Neutrophils by α-TREM-1 Promotes Wound Healing and Colitis

**Figure 6:**
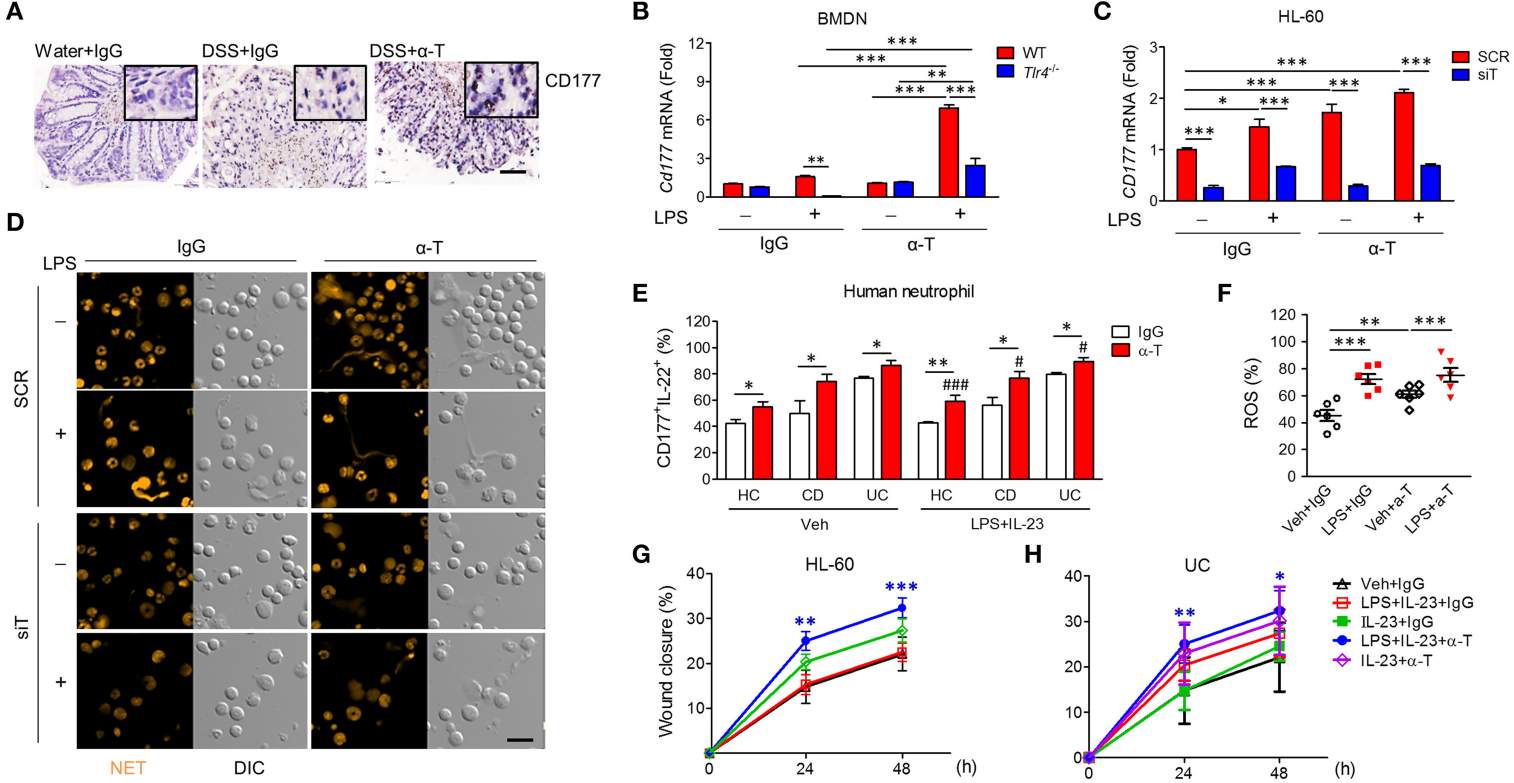
α-TREM-1 facilitates the cooperation of CD177^+^ neutrophils with intestinal epithelial cells and macrophages. **(A)** Representative images of CD177 immunostaining in colon tissues. Scale bar, 20 μm. **(B)** *Cd177* expression in mouse bone marrow-derived neutrophils (BMDNs) from wild-type and *Tlr4*-KO mice. BMDNs and HL-60 cells pre-treated with α-TREM-1 (0.8 μg/mL) were treated with lipopolysaccharide (LPS, 200 ng/mL) for 4 h for qRT-PCR analysis. Each data represents the mean of duplicate real-time RT-PCR (*n* = 3). **(C**,**D)** HL-60 cells transfected with scrambled (SCR) or *TREM1*-specific (siT) siRNA were pre-treated with α-TREM-1 and stimulated with LPS for 1 h. **(C)** *CD177* expression in human neutrophils treated with α-TREM-1. Each data represents the mean of duplicate real-time RT-PCR (*n* = 3). **(D)** Representative images from the neutrophil extracellular trap assay. Data are from one experiment representative of three independent experiments. Scale bar, 40 μm. **(E)** Modulation of CD177^+^IL-22^+^ neutrophils in the blood of inflammatory bowel disease (IBD) patients by α-TREM-1. Flow cytometric analysis was performed to evaluate the CD177^+^IL-22^+^ cells in neutrophil populations extracted from ulcerative colitis (UC) and Crohn's disease (CD) patients and healthy subjects (*n* = 5). Numbers indicate CD177^+^IL-22^+^ cell frequencies among neutrophils. **(F)** Reactive oxygen species (ROS) production in neutrophils of IBD patients. **(G**,**H)** Wound healing assay. HT-29 cells were cultured in conditioned media from HL-60 cells **(G)** or blood neutrophils of UC patients **(H)** after α-TREM-1 treatment and wound healing assays were performed. Data represent the average of four independent experiments. Statistical significance was assessed using one-way ANOVA followed by Tukey post-test. * and ^#^*P* < 0.05, **P < 0.01, *** and ^*###*^*P* < 0.005 vs. vehicle (Veh). Data are expressed as means ± S.E.M. (*n* = 4–5). α-T, treated with α-TREM-, treated with control antibody; Water, supplied with normal drinking water.

Recently, it was demonstrated that CD177^+^ neutrophils are functionally activated and negatively regulate IBD through IL-22 production (16). In agreement, we found that CD177 and IL-22 were markedly increased in the colons of α-TREM-1-treated mice (Figure 6A, Supplementary Figures 2B,C). After co-stimulation with LPS and α-TREM-1, we also observed an increase in *CD177* and *IL-22* expression in bone marrow-derived neutrophils (BMDNs) from WT mice as mouse primary neutrophil cells and in HL-60 cells as human neutrophil cells, but not in BMDNs from *Tlr4*-KO mice (Figure 6B, Supplementary Figure 10A) or in *TREM1*-knockdown HL-60 cells (Figure 6C, Supplementary Figure 10B). An additional pathogen-elimination mechanism was found for neutrophils in which they form neutrophil extracellular traps (NETs) (17). α-TREM-1-treated control HL-60 cells dramatically increased NET formation after LPS treatment, which was inhibited by *TREM1* knockdown (Figure 6D). Furthermore, α-TREM-1 treatment increased autophagy in HL-60 cells and BMDNs (Supplementary Figures 10C,D). To confirm that α-TREM-1 promotes CD177^+^ neutrophil differentiation in IBD, we examined the CD177^+^IL-22^+^ population after α-TREM-1 treatment of neutrophils from healthy controls and IBD patients, with or without LPS and IL-23. CD177^+^IL-22^+^ populations in neutrophils were significantly increased by α-TREM-1 treatment in both healthy controls and IBD patients (Figure 6E). Likewise, α-TREM-1 increased ROS production (Figure 6F) and LPS-induced *CD177, IL22*, and *TNFA* expression in neutrophils from IBD patients (Supplementary Figure 10E).

Next, we evaluated the role of α-TREM-1-treated neutrophils in intestinal barrier regulation, investigating whether increased wound healing occurs through IL-22 released by CD177^+^ neutrophils. Scraped colonic epithelial HT-29 cells were cultured in conditioned media from HL-60 cells treated with IgG or α-TREM-1 and various TLR ligands. HT-29 cells grown in media containing α-TREM-1-treated HL-60 cells and LPS+IL-23 showed greater wound closure than those grown in media containing Ig-G-treated HL-60 cells with LPS+IL-23 or LPS (Figure 6G, Supplementary Figure 10F). Like HL-60 cells, α-TREM-1-treated neutrophils from IBD patients promoted wound closure (Figure 6H, Supplementary Figure 10H).

**Figure 7:**
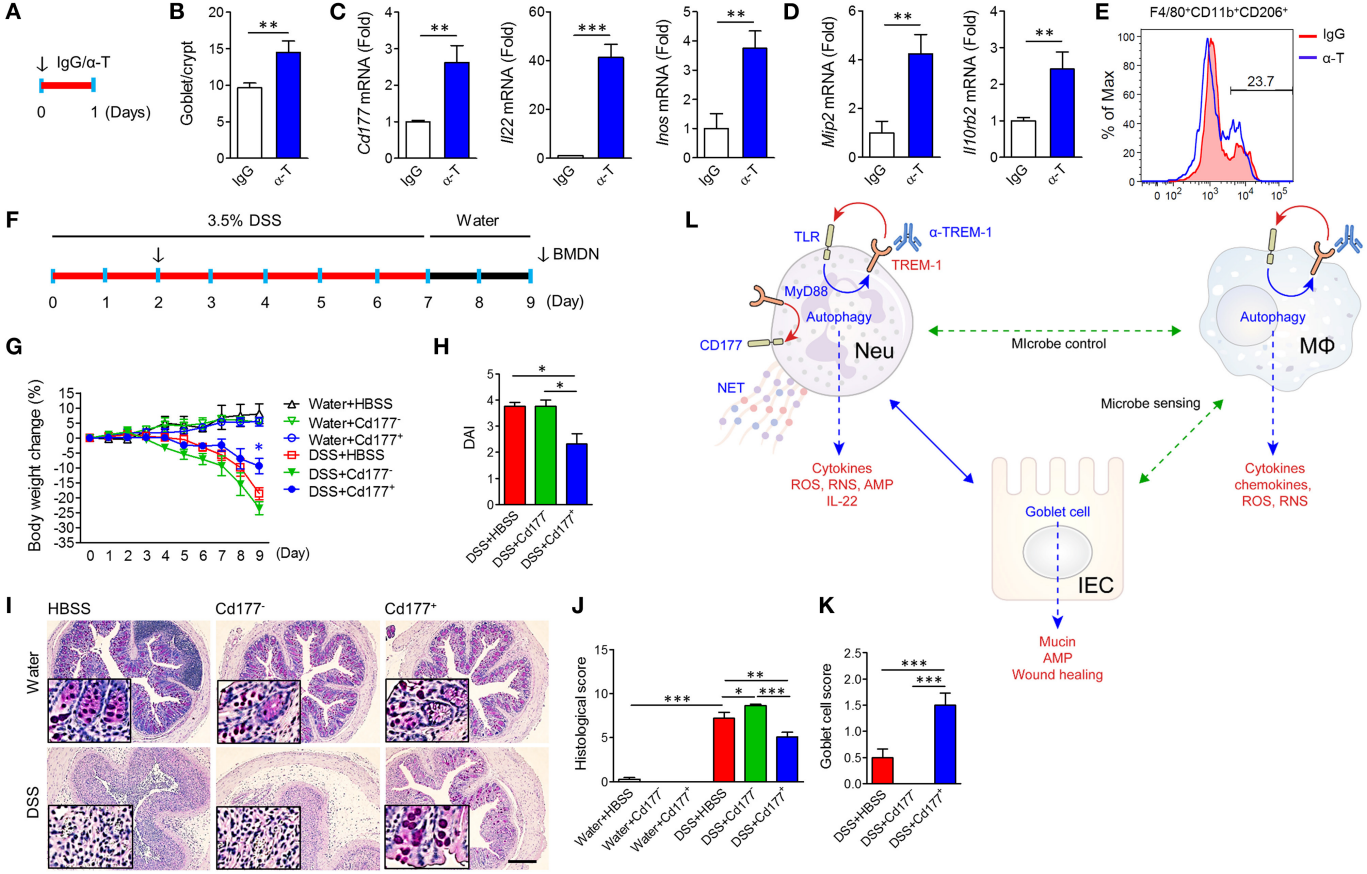
α-TREM-1-induced CD177^+^ neutrophils confer anticolitic effects. **(A–E)** α-TREM-1 was intraperitoneally injected to C57BL/6 mice and the next day, colons were isolated for analysis. **(A)** Experimental diagram. The arrow indicates the point at which IgG- or α-TREM-1 was administered (20 μg/mouse). **(B)** Goblet cell score. **(C,D)** Expression of Cd177^+^ neutrophil- (**C**: *Cd177, Il22*, and *Inos*) and macrophage-specific (**D**: *Mip2* and *Il10rb2*) genes in the colon. Each data represents the mean of duplicate real-time RT-PCR (*n* = 4). **(E)** Flow cytometric analysis of M2 (CD206^+^) in macrophage (F4/80^+^Cd11b^+^) populations among lamina propria mononuclear cells. **(F–J)** Bone marrow-derived neutrophils were isolated, treated with IgG or α-TREM-1 for 24 h, and sorted by FACS into CD177^+^ and CD177^−^, which were then intraperitoneally injected (1 × 10^6^ cells, arrow) into recipient mice 2 days after DSS treatment. **(F)** Experimental design for neutrophil transfer. **(G)** Body weight change. **(H)** Disease activity index. **(I)** Representative sections of periodic acid-Schiff staining. Scale bar, 200 μm. **(J)** Histological score. **(K)** Goblet cell score. **(L)** Schematic representation of anticolitic effects of α-TREM-1. Data are expressed as means ± S.E.M. (*n* = 4/groups). α-T, treated with α-TREM-1; AMP, antimicrobial peptides; IgG, treated with control antibody; HBSS, injected with Hank's balanced salt solution; IEC, intestinal epithelial cell; IgG, treated with control antibody; MΦ, macrophage; Neu, neutrophil; RNS, reactive nitrogen species; ROS, reactive oxygen species. Statistical significance was assessed using Student *t*-test **(B–D)** or one-way ANOVA followed by Tukey post-test **(G**,**H**,**J**,**K)**. *P < 0.05, **P < 0.01, ***P < 0.005.

We intraperitoneally injected α-TREM-1 (Figure 7A) and investigated changes in IECs and gene expression in colon tissues. α-TREM-1 administration markedly increased goblet cell numbers (Figure 7B) and expression of genes related to CD177^+^ neutrophils, macrophages, phagocytosis, and *Tlr4* (Figures 7C,D, Supplementary Figures 11A–D). M2 macrophage marker levels also increased in LPMCs after α-TREM-1 treatment (Figure 7E). To further confirm the role of CD177^+^ neutrophils in the anticolitic effects of α-TREM-1, we identified the differentiation of CD177^+^ BMDNs by α-TREM-1. We then prepared CD177^+^ and CD177^−^ BMDNs 1 day after α-TREM-1 treatment (Supplementary Figure 11E) and performed neutrophil transfer into mice on day 2 of DSS treatment (Figure 7F, Supplementary Figure 11E). CD177^+^ BMDN transfer markedly attenuated colitis and restored goblet cell numbers (Figures 7G–K, Supplementary Figure 11F). Flow cytometric analysis indicated that transferred CD177^+^ neutrophils migrated to the colon (Supplementary Figure 11G). These results highlight the importance of the TREM-1 pathway in CD177^+^ neutrophils, which leads to pathogen clearance and protection of the intestinal barrier in colitis.

## Discussion

IBD features neutrophil infiltration of the intestinal mucosa and repeated epithelial injury (1). However, neutrophils play essential roles in the innate immune response, resolution of inflammation, and healing processes in colitis (16, 18, 19). Indeed, a variety of defects in neutrophil function have been described in CD patients (20, 21). They play a critical bactericidal role as the first line using ROS/RNS intermediates, antimicrobial peptides, or NETs (22). In addition, the limited antimicrobial capacity of macrophages can be supplemented through the acquisition of neutrophilic microbicidal molecules or directly by neutrophil products, including released granule molecules (19, 23). Neutrophils from TREM-1-deficient mice have reduced ROS production, poor neutrophil recruitment, impaired pathogen clearance, and increased bacterial translocation and tissue damage in the intestine (8). In addition, CD177^+^ neutrophils have bactericidal activity and produce high levels of ROS, NET, IL-22, and antimicrobial peptides, and low levels of proinflammatory cytokines (16). These cells have an indispensable protective role in IBD (16), which is consistent with our data. Interestingly, CD177 is also an endogenous TREM-1 ligand (24), and CD177-deficiency leads to neutrophil death (25). Neutrophils can also enhance intestinal mucosal wound healing and barrier function through interactions with IECs (26). We showed that α-TREM-1 induced CD177^+^ neutrophils in the blood of IBD patients and healthy controls, and that α-TREM-1-driven CD177^+^ neutrophils ameliorate mouse colitis. These results suggest that CD177^+^ neutrophils limit inflammation through bactericidal activity and promote wound repair induced by IL-22 production, although our neutrophil depletion model can also induce opsonization and phagocytosis of targeted cells (27).

Pathogens stimulate pattern recognition receptors (PRR) via their pathogen-associated or damage-associated molecular patterns and induce proinflammatory mediators including TNF and IL-1β through NF-κB activation. This situation may mediate inflammation and help maintain gut barrier functions as a host defense mechanism (6, 15, 28). In contrast to studies on the deleterious roles of TREM-1 which had focused on blocking signaling (10), a large body of evidence suggests a beneficial role of TREM-1 agonists in bacterial clearance and infection resolution (7, 8, 29). Likewise, we found that co-administration of antibiotics with α-TREM-1 further reduced pathogenic bacterial load and markedly ameliorated colitis by significantly decreasing the proportion of *Proteobacteria*, which are Gram-negative bacteria that can induce colitis. Cohousing and FMT between α-TREM-1-treated mice and *Tlr4*-KO mice temporarily suppressed gut inflammation, suggesting that host immune response through TLR4 signaling is required for sustained effects. TLRs lead to important bactericidal activity through ROS generation (6), cytokine production, and IL-22 production in the colon (30). Consistently, *Tlr4*- and *Myd88*-KO mice are more susceptible to infection and colitis than WT mice due to increased bacterial translocation (31, 32) and decreased AMP expression (31). Likewise, we observed an increase in bacterial invasion and a decrease in the anticolitic effect of α-TREM-1 in *Tlr4*- and *Myd88*-KO mice, as well as high expression of inducible nitric oxide synthase (iNOS) and neutrophil ROS production in the colons of α-TREM-1-treated mice. Defects involving microbial sensing and bacterial handling pathways, including ROS production and antimicrobial autophagy, are also associated with enhanced risk of IBD (15). TREM-1 also cooperates with other PRRs and has synergistic effects on proinflammatory cytokine production (33); we have obtained corresponding results *in vitro*. However, our data showed that TLR4 signaling mainly mediates the anti-inflammatory effects of α-TREM-1. Intriguingly, a NOD2 ligand (MDP) and peptidoglycan (PGN) did not increase TREM-1 expression and LPS co-stimulation with a TLR1/2 agonist (PAM3CSK4) showed suppressive effects on iNOS, COX-2, and TREM-1 expression (Supplementary Figure 9G). Furthermore, suppression of TREM-1 by other TLR ligands such as FLG or PAM3 may be responsible for the suppression of wound healing (Supplementary Figure 10G). These data indicate that TREM-1 may selectively sense microbiota and respond accordingly, and thus may explain how NOD2 mutation influences bacterial handling in CD (15). Taken together, our data suggest that TLR4 signaling mediates the anti-inflammatory effects of α-TREM-1, and is an important TLR for pathogen control in IBD (34).

The innate immune response is pivotal as a primary defense against intestinal microbiota and provides initial resistance to invading pathogens (34). In this context, we showed that bacterial clearance with antibiotic pre-treatment alleviated colitis, suggesting that bacterial handling at the early stage is important for control of gut inflammation and microbiota modulation. We postulate that α-TREM-1 can control pathogens at the early stage of gut inflammation. Here, we also showed that α-TREM-1 enhanced phagocytosis and autophagy in macrophages and neutrophils, as reported in previous studies (35, 36). Macrophagic engulfment of apoptotic neutrophils is required for wound healing and ROS production (15, 37). Moreover, autophagy is required for the NETosis pathway in neutrophils (38), suggesting that autophagy is important for preventing bacterial spread. Recently, autophagy induction was suggested as a therapeutic strategy for IBD (39, 40). Contradictory to our results, a few studies using *Trem1*-KO mice reported the following: (1) TREM-1 deficiency can attenuate disease severity without affecting parasitic and viral infections; (2) TREM-1 deletion restores impaired autophagy (41); and (3) TREM-1 inhibition using LR12 peptide attenuates experimental colitis by restoring impaired autophagy (42). However, we cannot fully exclude the possibility of different mechanisms between TREM-1 deficiency and α-TREM-1. For example, we found that *Trem1*-KO mice had higher DAI values in normal condition without colitis (42). These contradictory results should be interpreted cautiously because agonists and antagonists may have different effects due to subtly different modes of action. Consistently, a recent study that TREM-1 loss exacerbates colitis in several mouse models solidifies our results (43).

As depicted in Figure 7L, α-TREM-1 modulated the bacterial clearance activity of macrophages and neutrophils and promoted the differentiation of neutrophils into CD177^+^ cells, leading to enhanced protection against both microbes and tissue damage. We speculate that the intrinsic modulatory mechanism of anti-TREM-1 antibody, including the alteration of macrophage function, is at least in part related with CD177+ neutrophils, although the mechanism of the anticolitic effect of α-TREM-1 is probably multi-factorial. Additionally, we can postulate that the upregulated TREM-1 levels in IBD may be due to impaired neutrophil function (19, 44), neutrophil recruitment (36, 45), or TLR signaling (46), but this requires further elucidation of the mechanisms by which α-TREM-1 alleviates colitis as well as its role in IBD pathogenesis. Moreover, we need further information on whether the function and differentiation of CD177^+^ neutrophils can be affected by genetic variations in patients with IBD and their effects on the function of macrophages, such as autophagic capability, ROS production, and M2 polarization.

This is the first study to demonstrate that stimulation of TREM-1 signaling using α-TREM-1 is effective at attenuating colitis. We showed that α-TREM-1 augmented bactericidal activity via reciprocal interactions between TLR4 and TREM-1, and improved wound healing via the interaction of macrophages, neutrophils, and the intestinal epithelial barrier. Furthermore, we identified α-TREM-1 as a candidate regulator of CD177^+^ neutrophils, which are pivotal players in achieving a balance between microbe control and tissue repair in the gut. Although immune-suppressive therapies such as anti-TNF agents are effective at ameliorating symptoms in some IBD patients, continued treatment increases susceptibility to infection (47). Further insights into the role of α-TREM-1 in IBD pathogenesis may provide a new therapeutic target for IBD.

## Data Availability Statement

The original contributions presented in the study are included in the article/Supplementary Material, further inquiries can be directed to the corresponding authors.

## Ethics Statement

The studies involving human participants were reviewed and approved by the Institutional Review Board of Severance Hospital, Yonsei University. The patients/participants provided their written informed consent to participate in this study. The animal study was reviewed and approved by the Institutional Animal Care and Use Committee of Yonsei University Severance Hospital, Seoul, Korea.

## Author Contributions

DS, XC, SWK, and JC designed the study and wrote the manuscript. DS, XC, SWK, DK, HM, and JK performed experiments. DS, XC, SWK, and SK were involved in data analysis. TK, WK, and JC were involved in sample acquisition. SWK and JC were involved in funding acquisition. All authors contributed to critical revision of the manuscript and approved the final version.

## Conflict of Interest

The authors declare that the research was conducted in the absence of any commercial or financial relationships that could be construed as a potential conflict of interest.
