# Supplementary material for: Triggering Receptor Expressed on Myeloid Cells-1 Agonist Regulates Intestinal Inflammation via Cd177+ Neutrophils
Source: Front Immunol. 2021 Mar 9;12:650864. doi: 10.3389/fimmu.2021.650864 (PMC7985452; doi:10.3389/fimmu.2021.650864)
Supplement: Supplementary file 1 [file Data_Sheet_1.PDF]

Supplementary Material

Supplementary Figures

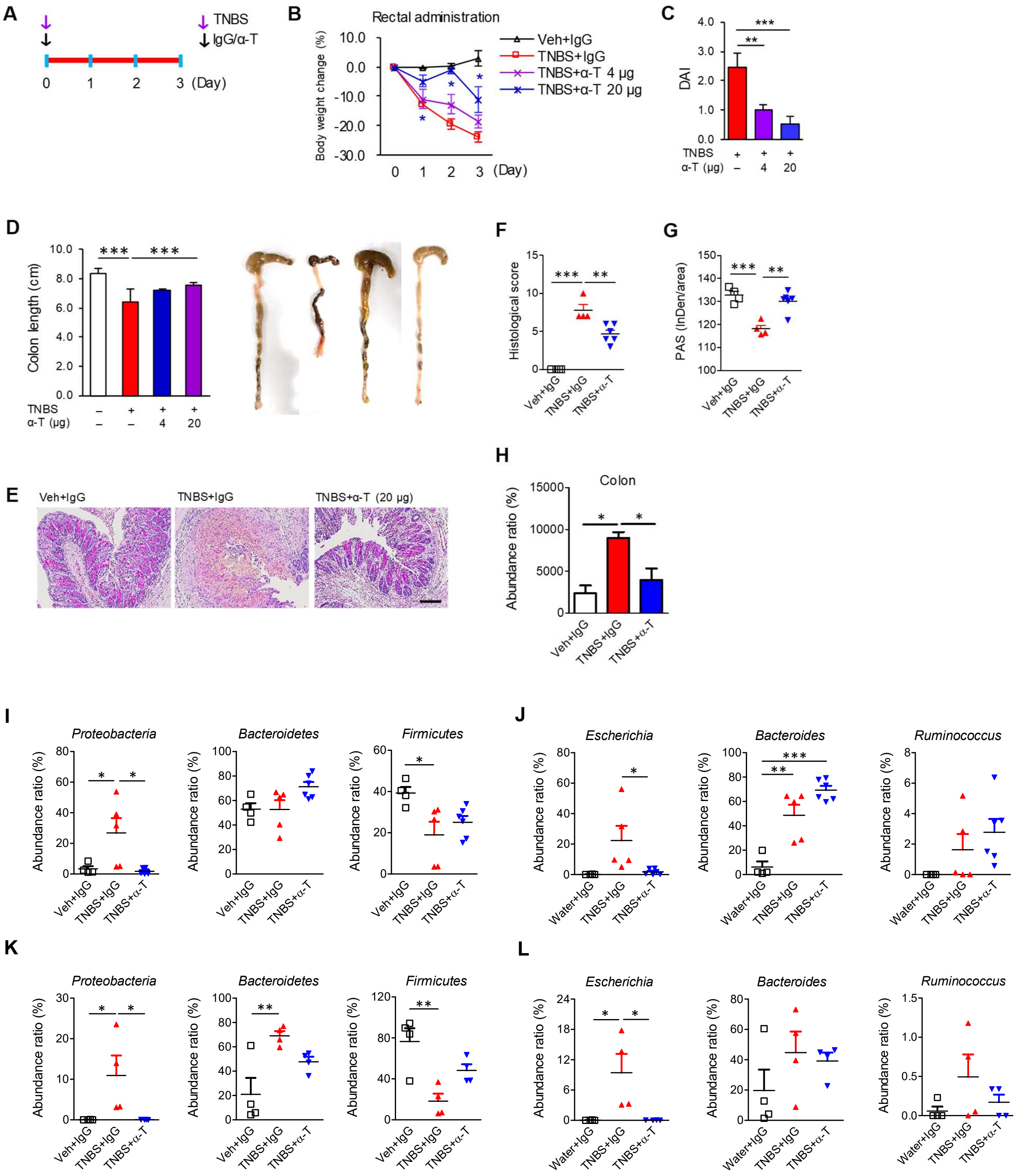

Supplementary Figure 1. Rectal administration of α-TREM-1 in TNBS-treated C57BL/6 mice. Colitis was evaluated after intrarectal injection of TNBS with control antibody (IgG) or α-TREM-1 (4 or 20 μg/mouse). (a) Experimental design. (b) Body weight change. (c) Disease activity index. (d) Colon length. (e) Representative sections of periodic acid-Schiff stain. Scale bar, 100 μm. (f) Histological score. (g) Goblet cell was assessed by PAS stain evaluation. (h) Bacterial loading in colon tissues. Genomic DNA isolated from colon tissues and the 16S rRNA gene was amplified, separated by electrophoresis, and products were analyzed using Image J software. (i, j) Microbiota profiles in colon tissues at phylum (i) and genus (j) level. (k, l) Microbiota profiles in the feces at the phylum (k) and genus (l) level. *$P < 0.05$, **$P < 0.01$, ***$P < 0.005$. Data are expressed as means ± S.E.M. (n = 4–8/groups). α-T, treated with TREM-1 antibody; TNBS, injected with trinitrobenzene sulfonic acid; Water, supplied with normal drinking water.

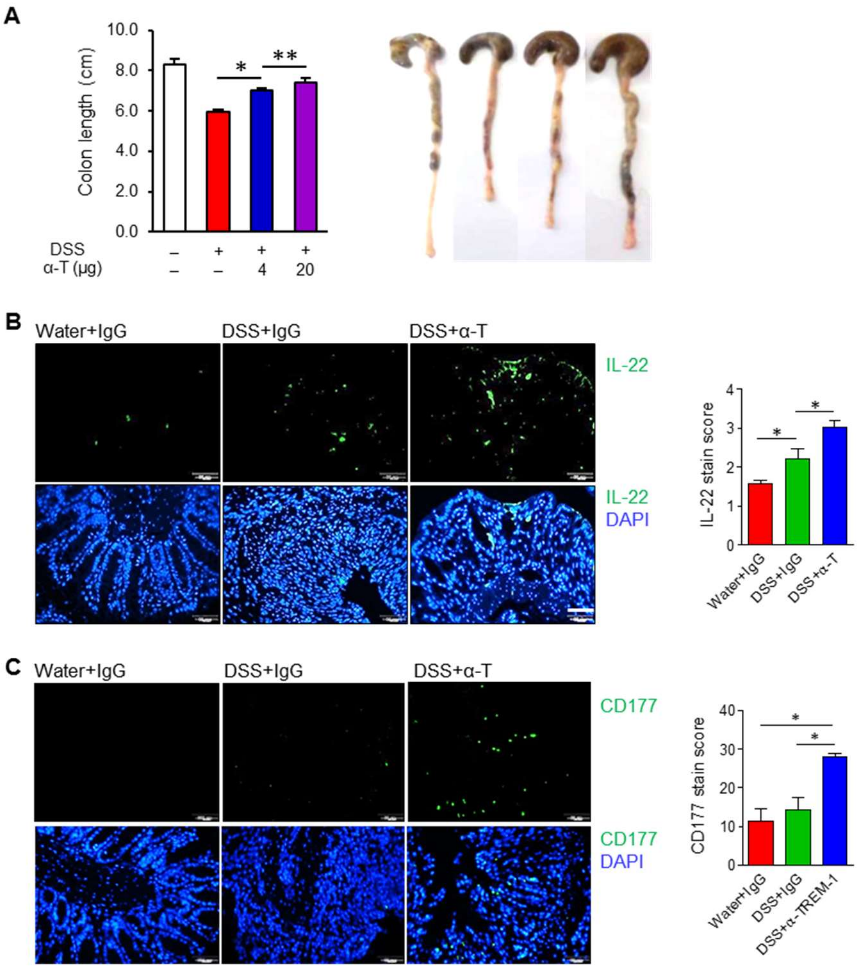

Supplementary Figure 2. Systemic administration of α-TREM-1 to DSS-treated mice. DSS was supplied in drinking water and an IgG or α-TREM-1 was intraperitoneally. injected into mice. (a) Colon length. (b, c) Representative sections (left) and scores (right) of IL-22 (b) and CD177 (c) staining. Scale bar, 20 μm. Data are expressed as means ± S.E.M. (n = 4–5). *$P < 0.05$, **$P < 0.01$, ***$P < 0.005$. Analyses were performed using one-way ANOVA with Tukey's posttest. α-T, treated with α-TREM-1; DSS, treated with dextran sulfate sodium; Water, supplied with normal drinking water.

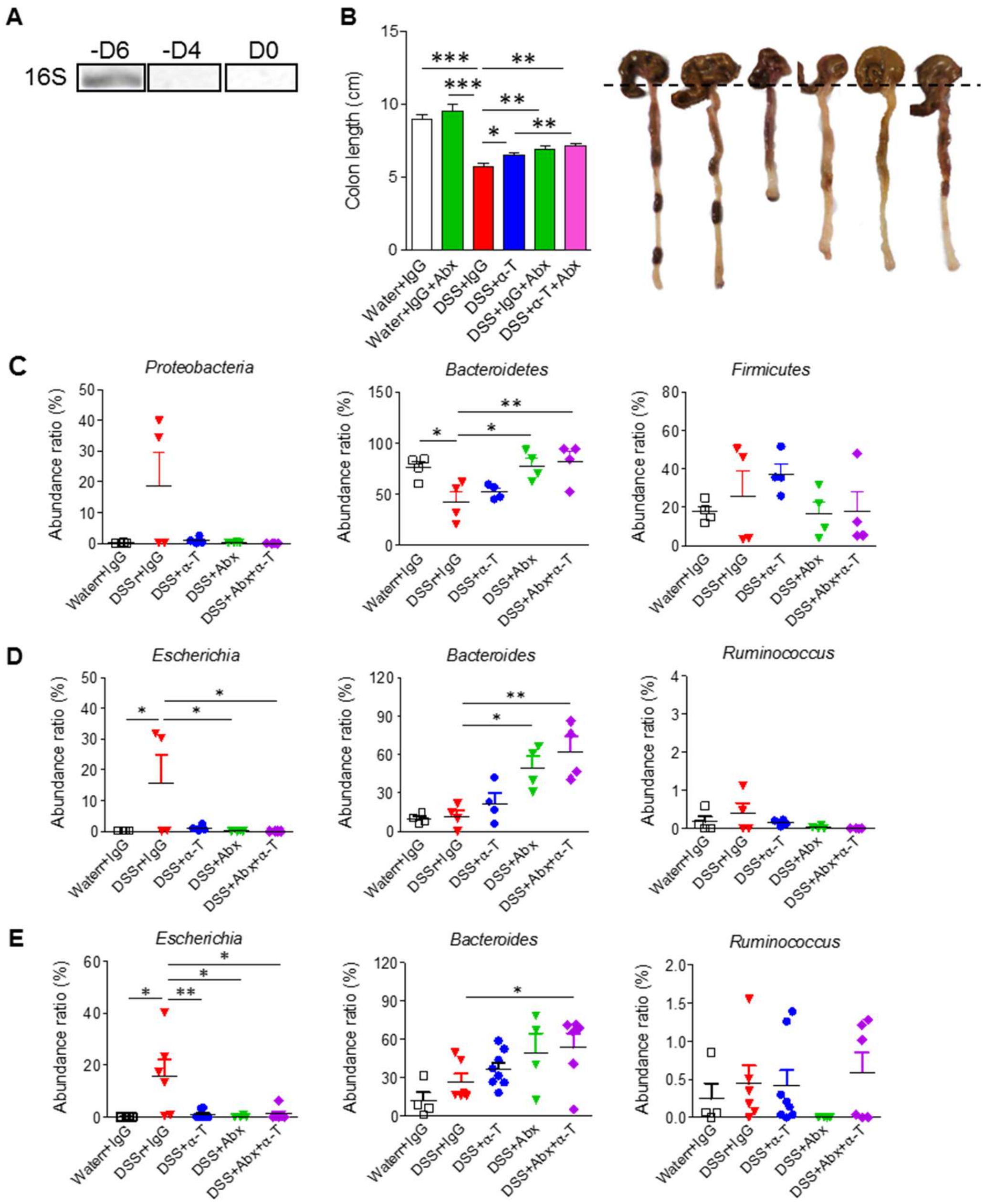

Supplementary Figure 3. Administration of α-TREM-1 and antibiotics to DSS-treated mice. An antibiotic cocktail (Abx) was supplied in drinking water 6 days before DSS administration (day 0) and IgG or α-TREM-1 was intraperitoneally injected into mice at day 7 (n = 8). (a) Bacterial load in the colon was assessed by 16S rRNA gene PCR. (b) Colon length. (c) Microbiota profiles in faeces at the phylum level. (d, e) Microbiota profiles in the feces (d) and colon (e) tissues at the

genus level. Data are expressed as means ± S.E.M. *$P < 0.05$, **$P < 0.01$, ***$P < 0.005$. Analyses were performed using one-way ANOVA with Tukey's posttest. α-T, treated with α-TREM-1; D, Day; DSS, treated with dextran sulfate sodium; Water, supplied with normal drinking water.

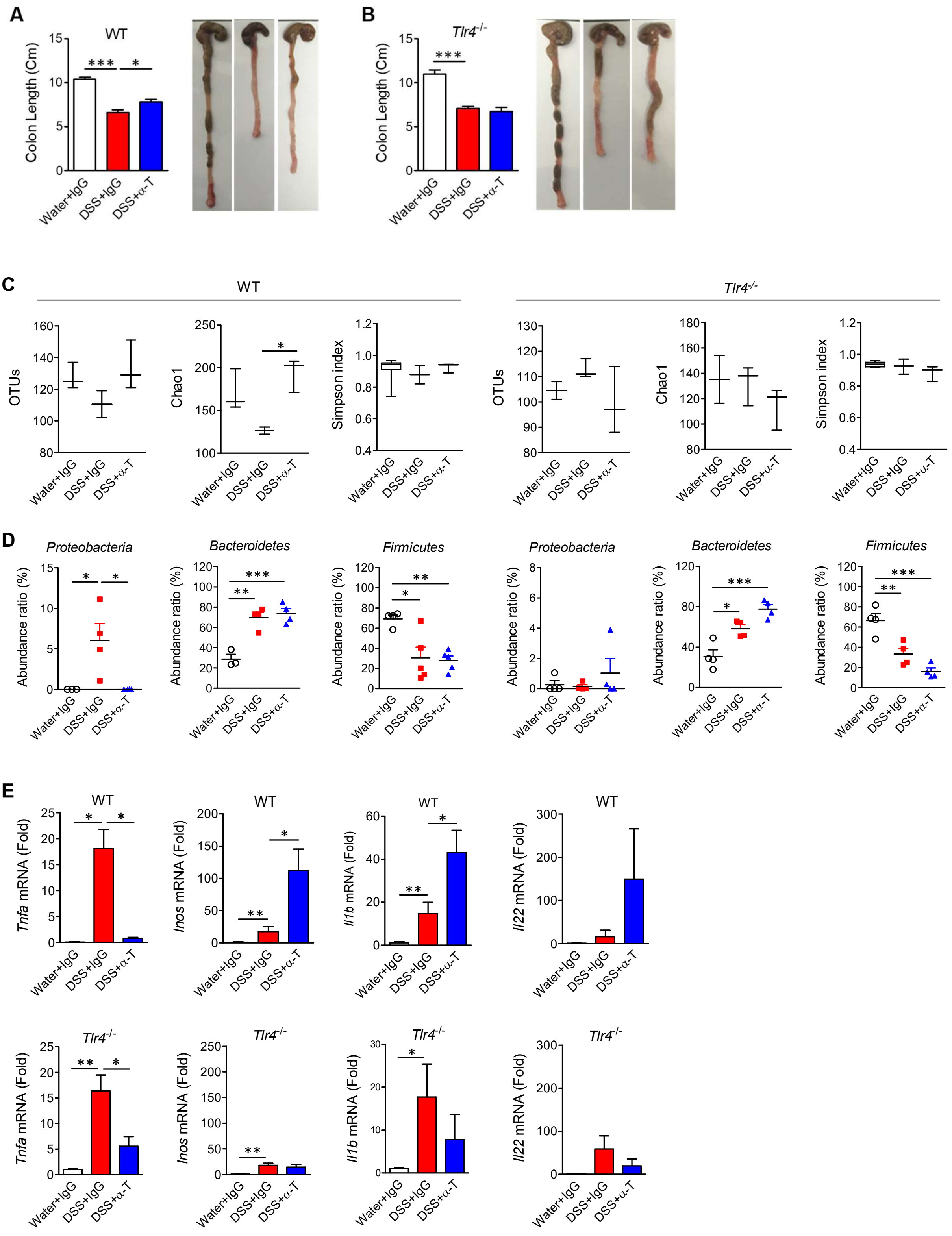
A
WT
Colon Length (Cm)
Water+IgG
DSS+IgG
DSS+α-T
B
Tlr4-/-
C
WT
Tlr4-/-
OTUs
Chao1
Simpson index
D
Proteobacteria
Bacteroidetes
Firmicutes
Abundance ratio (%)
E
Tnfa mRNA (Fold)
Inos mRNA (Fold)
Il1b mRNA (Fold)
Il22 mRNA (Fold)

Supplementary Figure 4. Administration of α-TREM-1 to DSS-treated wild-type and *Tlr4*-knockout mice. Wild-type (WT) and *Tlr4*-knockout ($Tlr4^{-/-}$) mice were subjected to a colitis and healing model with 3.5% DSS treatment for 8 days and normal drinking water for 2 days. IgG or α-TREM-1 was administrated once at day 2 (n = 7). (a, b) Colon length of WT and *Tlr4*-KO mice. (c) Total number of bacterial OTUs (left), richness predicted by the Chao1 index (middle), and diversity by the Shannon index (right) in the feces. (d) Microbiota profiles in the feces at the phylum level. (e) *Tnfa*, *Inos*, *Il1b*, and *Il22* expression profiles in colons of WT and *Tlr4*-KO mice with colitis as evaluated by qRT-PCR. Data are expressed as means ± S.E.M. (n = 7). *$P < 0.05$, **$P < 0.01$, ***$P < 0.005$. Analyses were performed using one-way ANOVA with Tukey's posttest. α-T, treated with α-TREM-1; DSS, treated with dextran sulfate sodium; Water, supplied with normal drinking water.

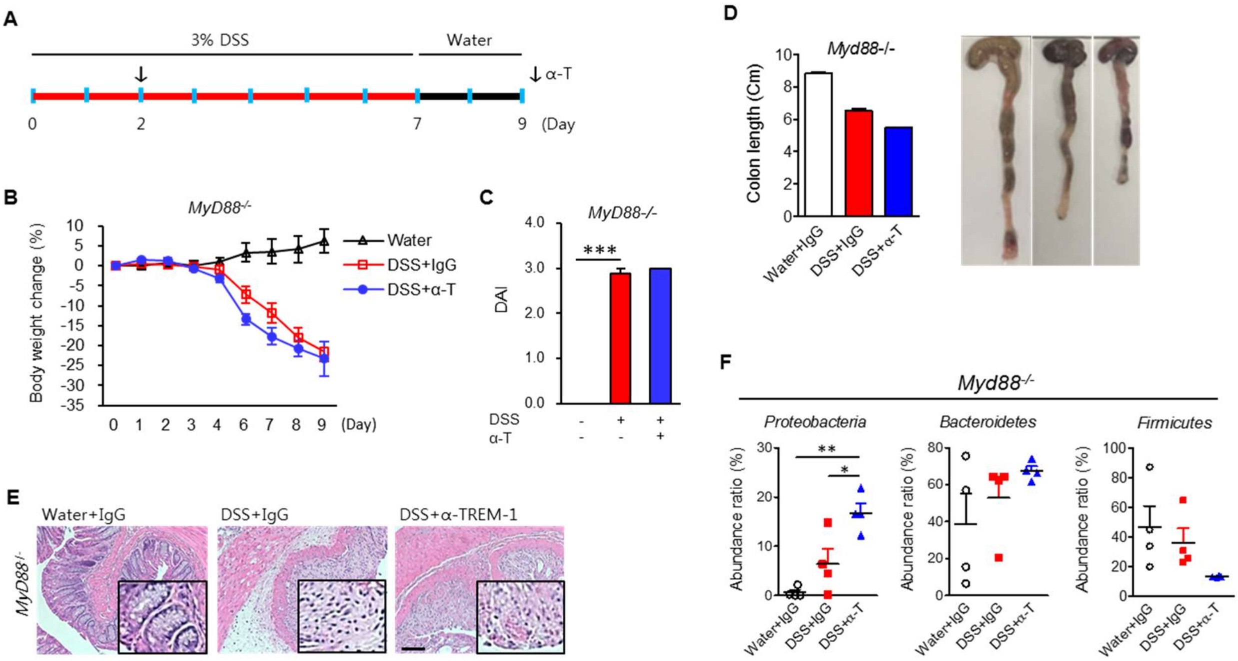

Supplementary Figure 5. Administration of α-TREM-1 to DSS-treated *Myd88*-knockout mice. Wild-type (WT) and *Myd88*-knockout (*Myd88*$^{-/-}$) mice were subjected to a colitis and healing model with 3.5% DSS treatment for 8 days and normal drinking water for 2 days, with a single administration of IgG or α-TREM-1 at day 2. (a) Experimental design. (b) Body weight change. (c) Disease activity index. (d) Colon length. (e) Representative sections of periodic acid-Schiff (PAS) stain. Scale bar, 20 μm. (f) Microbiota profiles in the colon at the phylum level. Data are expressed as means ± S.E.M. (n = 4-7). **P* < 0.05, ***P* < 0.01, ****P* < 0.005. Analyses were performed using one-way ANOVA with Tukey's posttest. α-T, treated with α-TREM-1; DSS, treated with dextran sulfate sodium; Water, supplied with normal drinking water.

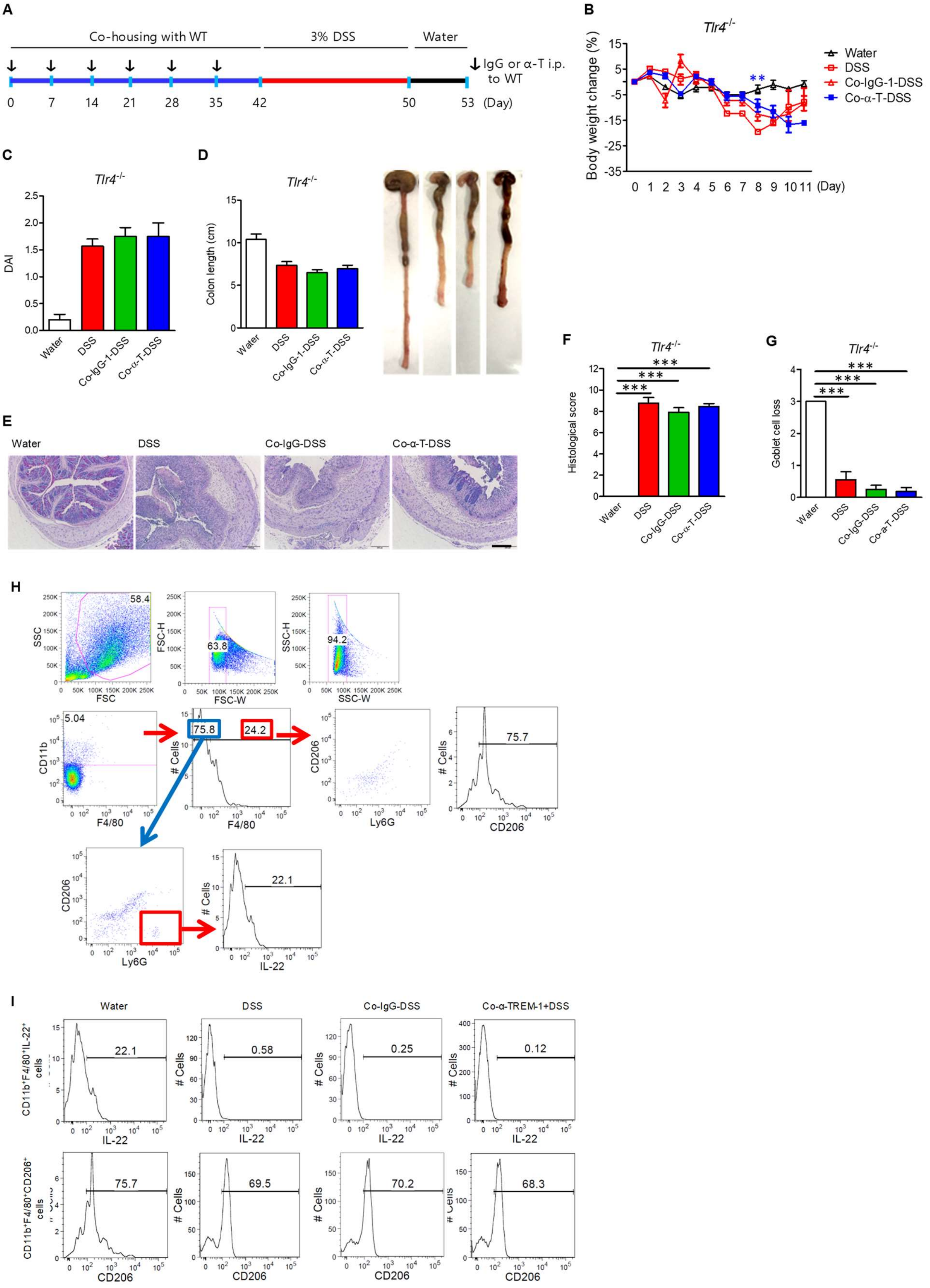
A
Co-housing with WT
3% DSS
Water
IgG or α-T i.p. to WT
0
7
14
21
28
35
42
50
53
(Day)
B
Tlr4-/-
Body weight change (%)
15
5
-5
-15
-25
-35
0 1 2 3 4 5 6 7 8 9 10 11 (Day)
**
Water
DSS
Co-IgG-1-DSS
Co-α-T-DSS
C
Tlr4-/-
DAI
2.5
2.0
1.5
1.0
0.5
0.0
Water
DSS
Co-IgG-1-DSS
Co-α-T-DSS
D
Tlr4-/-
Colon length (cm)
15
10
5
0
Water
DSS
Co-IgG-1-DSS
Co-α-T-DSS
E
Water
DSS
Co-IgG-DSS
Co-α-T-DSS
F
Tlr4-/-
Histological score
***
***
***
12
10
8
6
4
2
0
Water
DSS
Co-IgG-DSS
Co-α-T-DSS
G
Tlr4-/-
Goblet cell loss
***
***
***
4
3
2
1
0
Water
DSS
Co-IgG-DSS
Co-α-T-DSS
H
SSC
FSC
58.4
FSC-H
FSC-W
63.8
SSC-H
SSC-W
94.2
CD11b
F4/80
5.04
Cells
F4/80
75.8
24.2
CD206
Ly6G
Cells
CD206
75.7
CD206
Ly6G
Cells
IL-22
22.1
I
Water
DSS
Co-IgG-DSS
Co-α-TREM-1+DSS
CD11b+F4/80+IL-22+ cells
Cells
IL-22
22.1
0.58
0.25
0.12
CD11b+F4/80+CD206+ cells
CD206
75.7
69.5
70.2
68.3

Supplementary Figure 6. Effects on *Tlr4*-KO mice after cohousing with α-TREM-1-treated wild-type mice. *Tlr4*-KO mice were cohoused with wild-type mice treated with IgG or α-TREM-1 6 times for 6 weeks and treated with 3% DSS for 8 days and then untreated drinking water for 3 days (n = 4). (a) Experimental design. (b) Body weight change. (c) Disease activity index. (d) Colon length. (e) Representative sections of periodic acid-Schiff stain. Scale bar, 200 μm. (f) Histological score. (g) Goblet cell score. (h, i) Flow cytometry analysis of peritoneal cavity cells. (h) Gating strategy of flow cytometry. (i) Flow cytometric analysis of IL-22-producing and M2 macrophage ($CD206^+$) populations. Results are representative of 3 independent experiments. Data are expressed as means ± S.E.M. **P* < 0.05, ***P* < 0.01, ****P* < 0.005. Analyses were performed using one-way ANOVA with Tukey's posttest. α-T, treated with α-TREM-1; DSS, treated with dextran sulfate sodium; Water, supplied with normal drinking water.

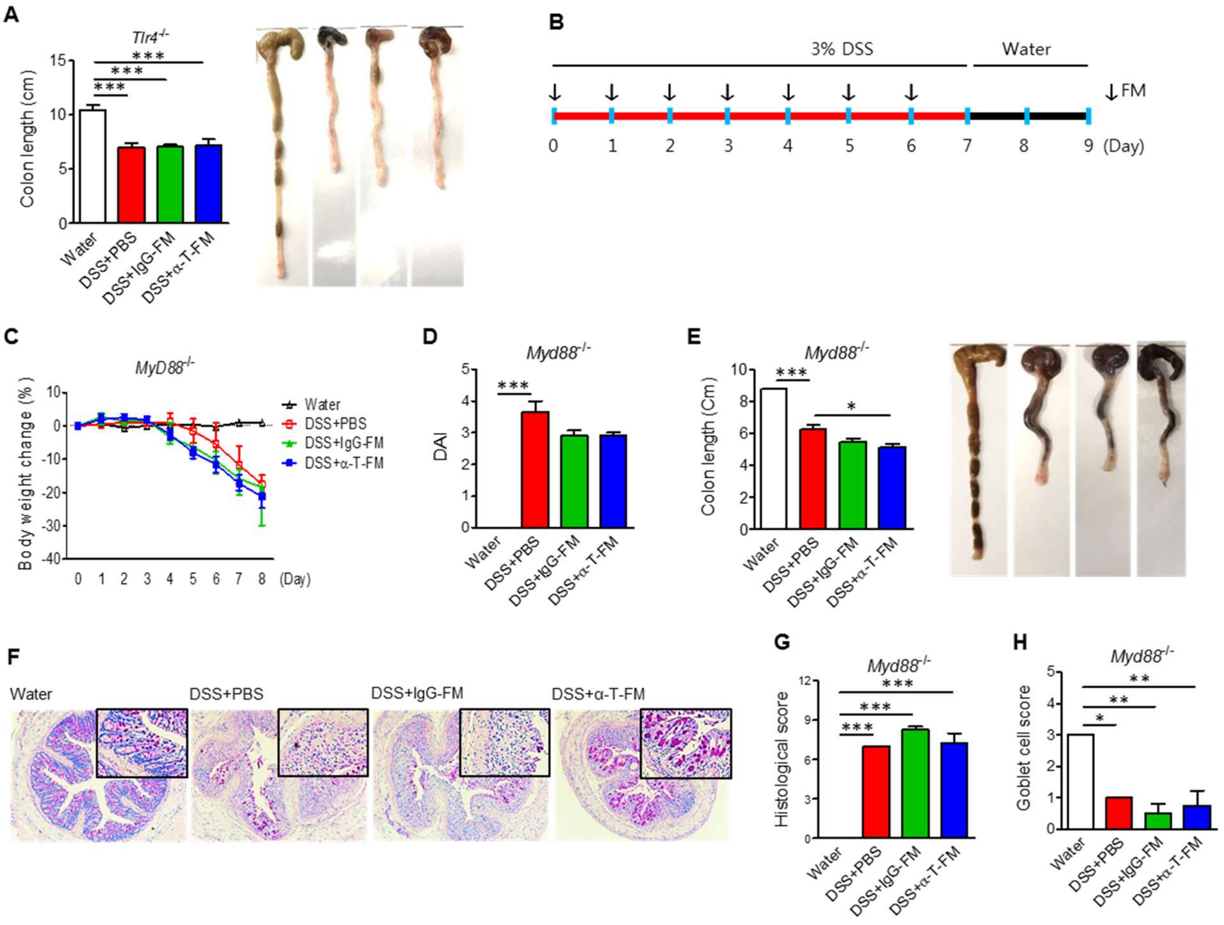

Supplementary Figure 7. Fecal microbiota transplantation of α-TREM-1-wild-type mice. (a) Colon length of fecal microbiota (FM)-transplanted *Tlr4*-KO mice. WT mice were intraperitoneally treated with IgG- and α-TREM-1 6 times weekly and the feces of mice were collected every day. Mice were orally administered FM of IgG- or α-TREM-1-treated mice daily for 8 days after 3% DSS treatment and DSS was then exchanged with drinking water for 2 days until the endpoint of the experiment (n = 5). (b–h) FM transplantation to *Myd88*-KO mice. WT mice were intraperitoneally treated with IgG- and α-TREM-1 6 times weekly and the feces of mice were collected every day. *Myd88*-KO Mice were orally administered FM of α-TREM-1-treated mice daily for 7 days after 3% DSS treatment and DSS was then exchanged with drinking water for 2 days until the endpoint of the experiment. (b) Experimental design. (c) Body weight

change. (d) Disease activity index. (e) Colon length. (f) Periodic acid-Schiff (PAS) stain. (g) Histological score. (h) Goblet cell score. Data are expressed as means ± S.E.M. (n = 4). **P* < 0.05, ***P* < 0.01, ****P* < 0.005. Analyses were performed using one-way ANOVA with Tukey's posttest. α-T, treated with α-TREM-1; DSS, treated with dextran sulfate sodium; Water, supplied with normal drinking water.

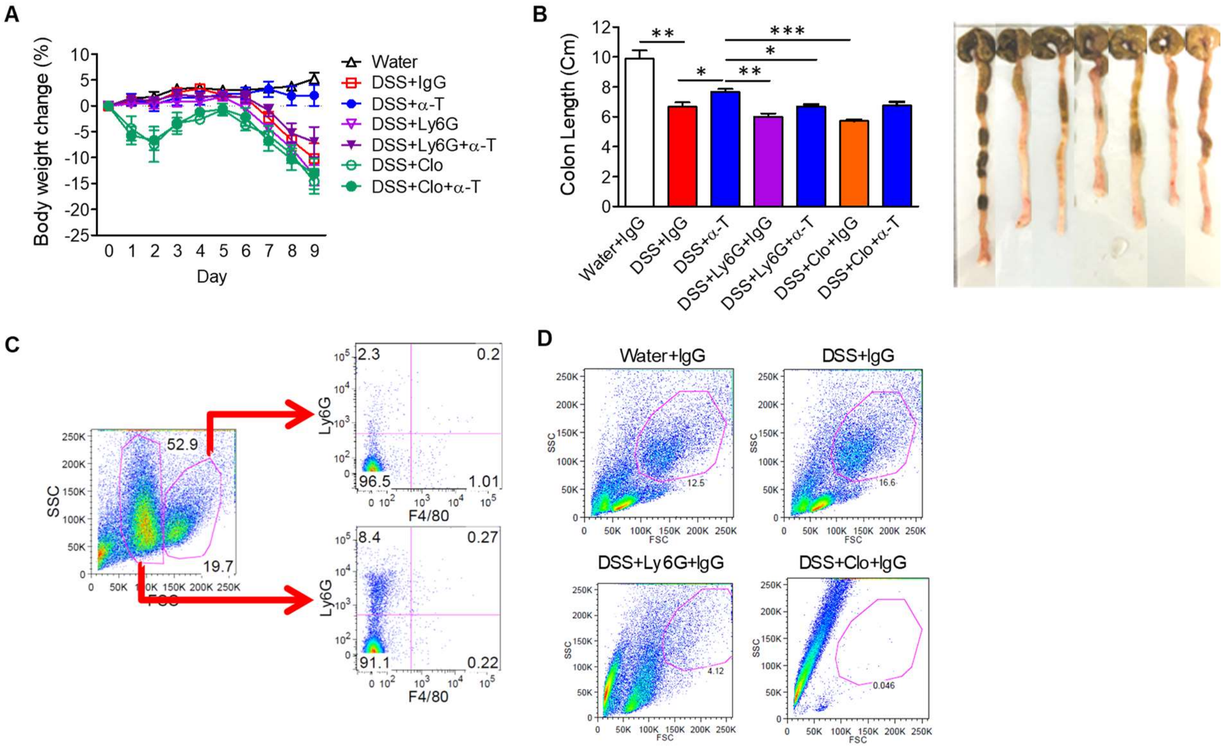

Supplementary Figure 8. Neutrophils and macrophages are required for the anticolitic effects of α-TREM-1. Mice were intraperitoneally administered twice (at day 1 and 2) with an anti-Gr1 antibody (Ly6G) to deplete neutrophils or twice (at day 1 and 6) with clodronate (Clo) to deplete macrophages and once with IgG or α-TREM-1 at day 2. After 3.5% DSS treatment for 8 days, DSS was exchanged with drinking water for 2 days (n = 4–6). (a) Body weight change. (b) Colon length. (c) Gating strategy for flow cytometric analysis of lamina propria mononuclear cells. (d) Flow cytometric analysis of lamina propria mononuclear cells. **P* < 0.05, ***P* < 0.01, ****P* < 0.005. Analyses were performed using one-way ANOVA with Tukey's posttest. α-T, treated with α-TREM-1; DSS, treated with dextran sulfate sodium; Water, supplied with normal drinking water.

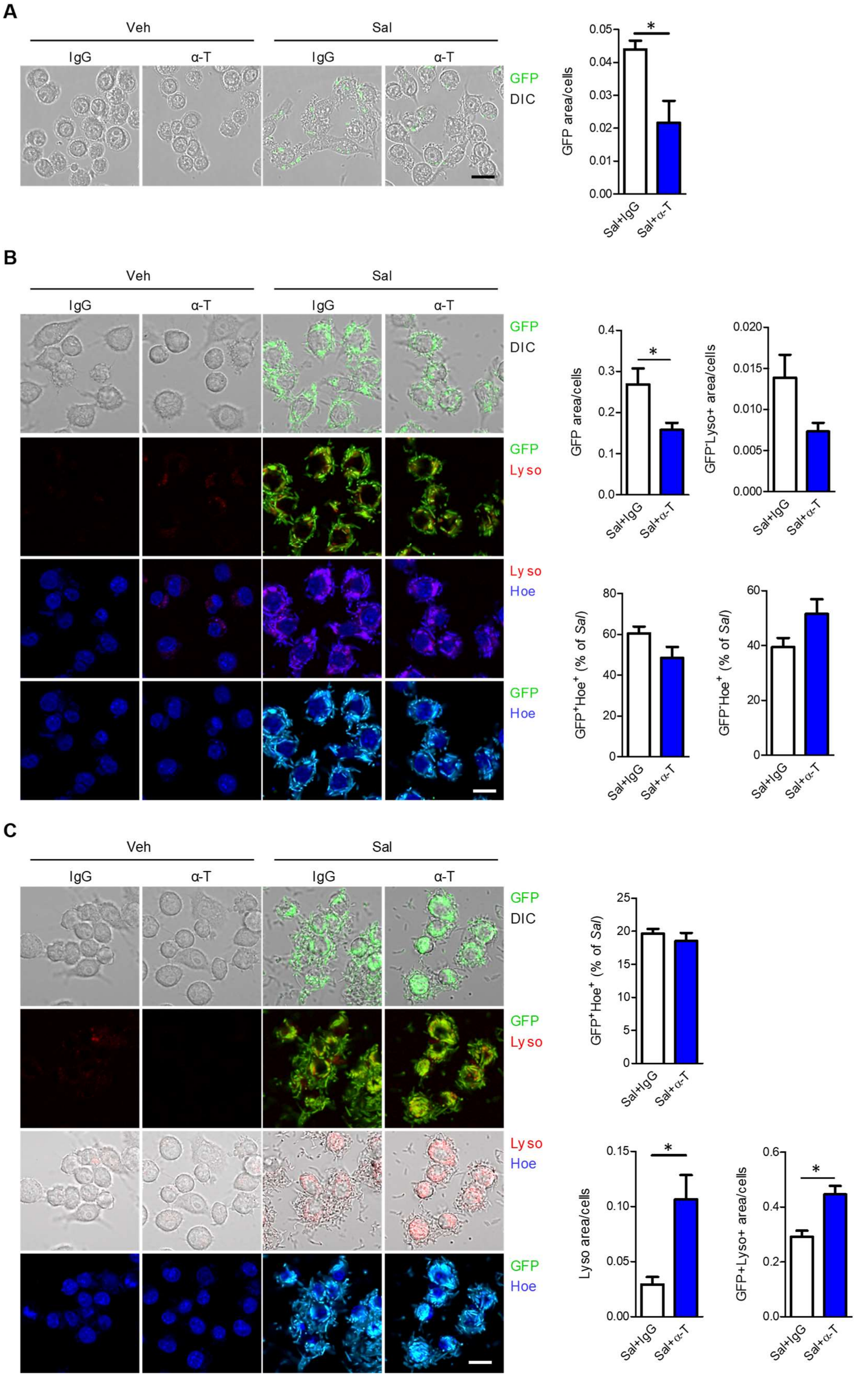
A
Veh
Sal
IgG
α-T
IgG
α-T
GFP
DIC
GFP area/cells
0.05
0.04
0.03
0.02
0.01
0.00
*
Sal+IgG
Sal+α-T
B
Veh
Sal
IgG
α-T
IgG
α-T
GFP
DIC
GFP
Lyso
Lyso
Hoe
GFP
Hoe
GFP area/cells
0.4
0.3
0.2
0.1
0.0
*
Sal+IgG
Sal+α-T
GFP⁻Lyso+ area/cells
0.020
0.015
0.010
0.005
0.000
Sal+IgG
Sal+α-T
GFP⁺Hoe⁺ (% of Sal)
80
60
40
20
0
Sal+IgG
Sal+α-T
GFP⁻Hoe⁺ (% of Sal)
60
40
20
0
Sal+IgG
Sal+α-T
C
Veh
Sal
IgG
α-T
IgG
α-T
GFP
DIC
GFP
Lyso
Lyso
Hoe
GFP
Hoe
GFP⁺Hoe⁺ (% of Sal)
25
20
15
10
5
0
Sal+IgG
Sal+α-T
Lyso area/cells
0.15
0.10
0.05
0.00
*
Sal+IgG
Sal+α-T
GFP+Lyso+ area/cells
0.6
0.4
0.2
0.0
*
Sal+IgG
Sal+α-T

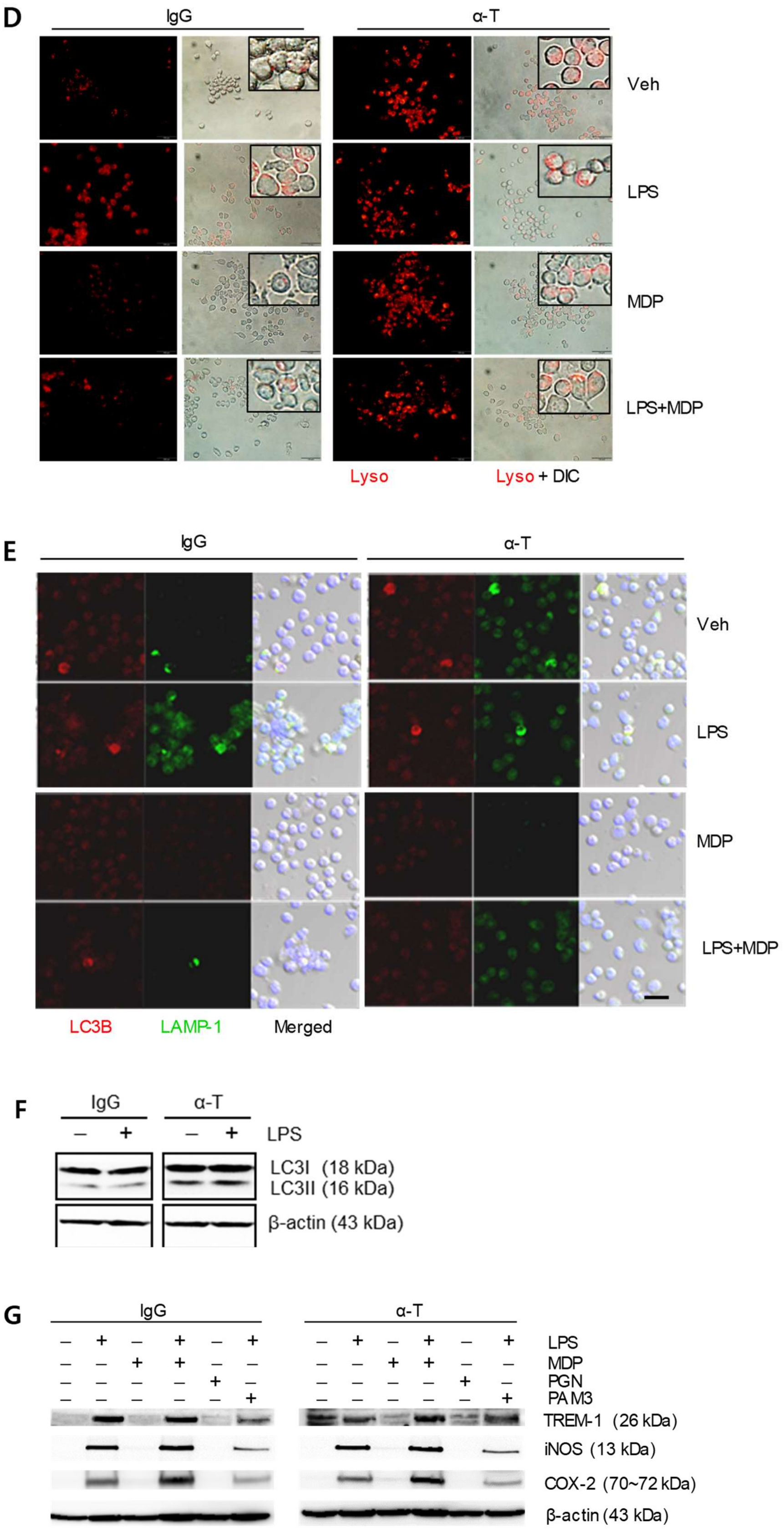
D
IgG
α-T
Veh
LPS
MDP
LPS+MDP
Lyso
Lyso + DIC
E
IgG
α-T
Veh
LPS
MDP
LPS+MDP
LC3B
LAMP-1
Merged
F
IgG
α-T
– + – + LPS
LC3I (18 kDa)
LC3II (16 kDa)
β-actin (43 kDa)
G
IgG
α-T
LPS
MDP
PGN
PAM3
TREM-1 (26 kDa)
iNOS (13 kDa)
COX-2 (70~72 kDa)
β-actin (43 kDa)

Supplementary Figure 9. α-TREM-1 promotes macrophage function. (a–c) RAW264.7 cells were pretreated with IgG or α-TREM-1 and infected with *S. typhimurium*-GFP (A, MOI 10 for 1 h; B, MOI 100 for 1 h; C, MOI 100 for 5 h). (a) Representative images of *S. typhimurium*-GFP-infected macrophages after infection for 1 h and quantification of GFP area per macrophage (right). Scale bar, 40 μm. (b) Representative images of *S. typhimurium*-GFP-infected macrophages after infection for 1 h and percentage of live (live *S. typhimurium*: GFP area and $GFP^{+}Hoechst^{+}$) or dead ($GFP^{-}Hoechst^{+}$) *S. typhimurium*-GFP and *S. typhimurium*-GFP in autophagic degradation (GFP co-localization with lysosomes, $GFP^{-}Lyso^{+}$ area). Scale bar, 20 μm. (c) Representative images of *S. typhimurium*-infected macrophages after infection for 5 h and percentage of live (live *S. typhimurium* GFP: GFP area and $GFP^{+}Hoechst^{+}$), lysosome (Lyso area), and *S. typhimurium*-GFP in autophagic degradation ($GFP^{+}Lyso^{+}$ area). Scale bar, 20 μm. (d) Evaluation of lysosomes (lysotracker stain) using fluorescence microscopy in cells stimulated with lipopolysaccharide (LPS, 200 ng/mL) and muramyl dipeptide (MDP, 10 μg/mL) for 4 h. Scale bar, 40 μm. (e) Evaluation of autophagosomes (LAMP-1 and LC3B staining) using confocal fluorescence microscopy in cells stimulated with LPS and MDP for 16 h. Scale bar, 40 μm. (f) Western blotting for LC3B and β-actin. RAW264.7 cells were stimulated with lipopolysaccharide (LPS, 200 ng/mL) for 8 h after pre-treatment with α-TREM-1 (0.8 μg/mL). (g) Western blotting of TREM-1, iNOS, COX-2, and β-actin. RAW264.7 cells were stimulated with LPS (200 ng/mL), muramyl dipeptide (MDP, 10 μg/mL), peptidoglycan (PGN, 1 μg/mL), or Pam3CSK4 (PAM3, 100 ng/mL) for 12 h after pretreatment with α-TREM-1 (0.8 μg/mL). Data are expressed as means ± S.E.M. (n = 4–5). α-T, treated with α-TREM-1; DIC, differential interference contrast; Hoec, Hoechst; Lyso, lysotracker; MOI, multiplicity of infection; *Sal*, infected with *S. typhimurium*-GFP; Veh, treated

with vehicle. *$P < 0.05$, **$P < 0.01$, ***$P < 0.005$. Analyses were performed using one-way ANOVA with Tukey's posttest.

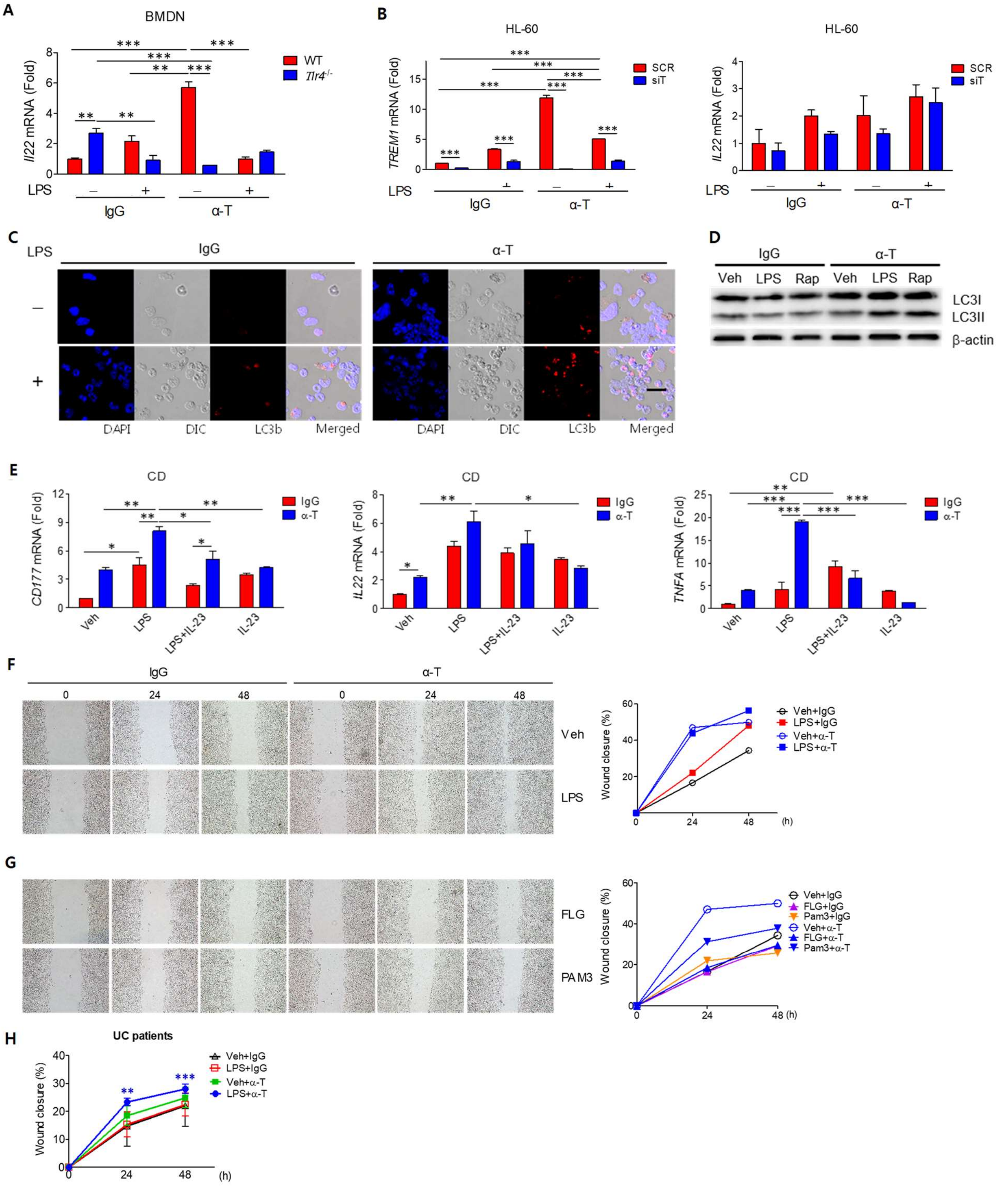

Supplementary Figure 10. α-TREM-1 promotes neutrophil function. Bone marrow-derived neutrophils (BMDNs) or HL-60 cells were pretreated with α-TREM-1 (0.8 μg/mL) and then

treated with lipopolysaccharide (LPS, 200 ng/mL), flagellin (FLG, 200 ng/mL), or Pam3CSK4 (PAM3, 100 ng/mL) for 24 h for flow cytometric analysis, western blotting analysis, and wound healing assays, or for 4 h for qRT-PCR analysis, otherwise for the indicated time periods. (a) *Il22* expression in BMDNs of wild-type (WT) and *Tlr4*-KO mice. (b) *TREM1* and *IL22* gene expression. HL-60 cells transfected with scrambled (SCR) or *TREM1*-specific (siT) siRNA were pretreated with α -TREM-1 and then stimulated with LPS for 1 h. (c) Representative images of LC-3B-stained HL-60 cells. Scale bar, 40 μm. HL-60 cells were stimulated with LPS for 4 h after pretreatment with α-TREM-1. (d) Autophagy activation was evaluated using western blotting of LC-3B in BMDNs. BMDNs were treated with rapamycin (100 nM). (e) *CD177*, *IL22*, and *TNFA* expression in neutrophils of patients with Crohn's disease. (f–h) Wound healing assay. (f, g) Culture media of wounded HT-29 cells were exchanged with conditioned media of HL-60 cells after stimulation with LPS (f), FLG, or PAM3 (g) and co-stimulation with α-TREM-1. (h) Culture media of wounded HT-29 cells were exchanged with conditioned media of LPS-treated blood neutrophils from ulcerative colitis patients. Data are expressed as means ± S.E.M. (n = 3–6). *$P < 0.05$, **$P < 0.01$, ***$P < 0.005$. Analyses were performed using one-way ANOVA with Tukey's posttest. α-T, treated with α-TREM-1; DAPI, 4′, 6-diamidino-2-phenylindole; DIC, differential interference contrast; Veh, treated with vehicle.

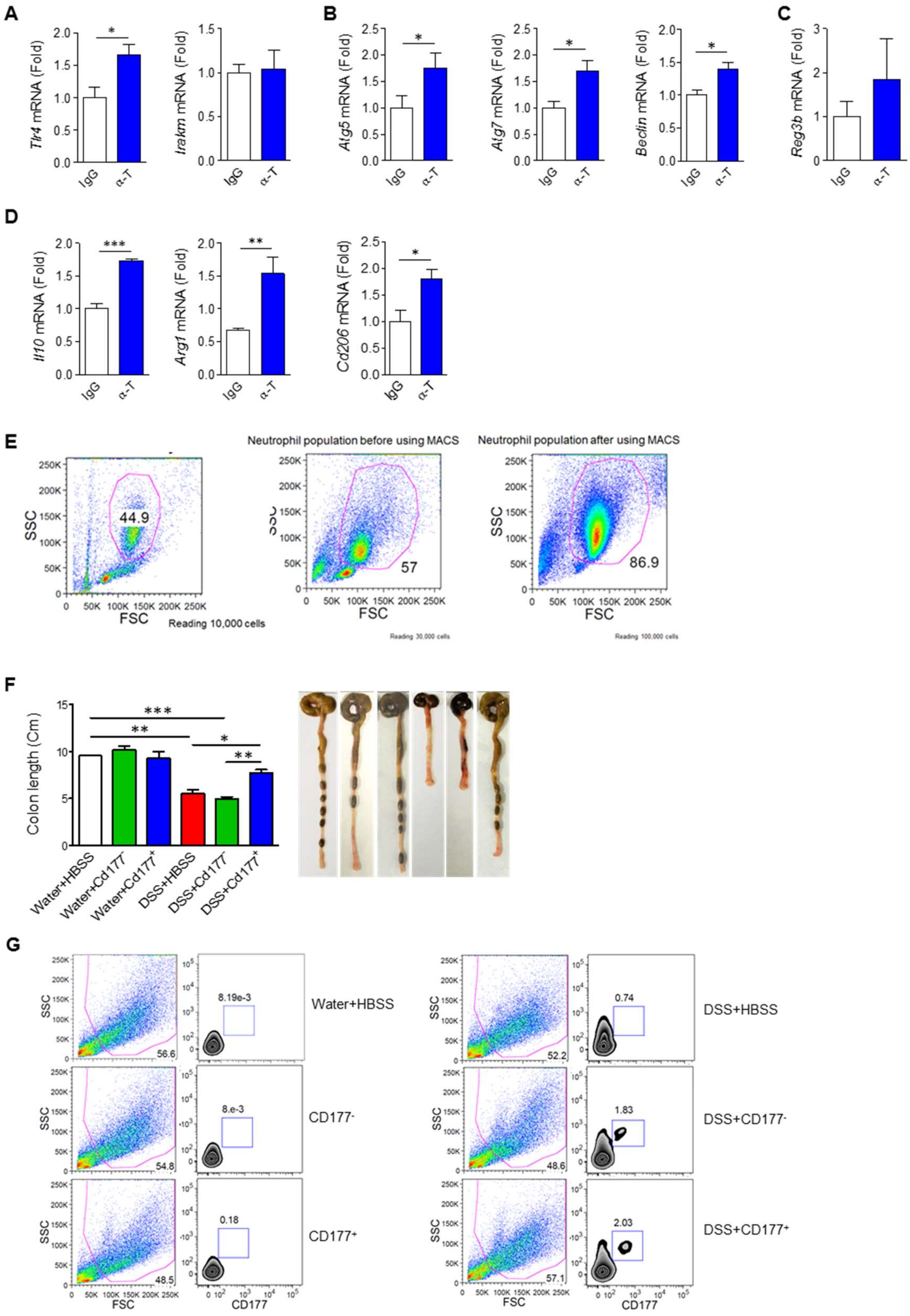
A
Tlr4 mRNA (Fold)
Irakm mRNA (Fold)
IgG
α-T
B
Atg5 mRNA (Fold)
Atg7 mRNA (Fold)
Beclin mRNA (Fold)
C
Reg3b mRNA (Fold)
D
Il10 mRNA (Fold)
Arg1 mRNA (Fold)
Cd206 mRNA (Fold)
E
Neutrophil population before using MACS
Neutrophil population after using MACS
SSC
FSC
44.9
57
86.9
Reading 10,000 cells
F
Colon length (Cm)
Water+HBSS
Water+Cd177⁻
Water+Cd177⁺
DSS+HBSS
DSS+Cd177⁻
DSS+Cd177⁺
G
Water+HBSS
CD177⁻
CD177⁺
DSS+HBSS
DSS+CD177⁻
DSS+CD177⁺
8.19e-3
8.e-3
0.18
0.74
1.83
2.03
56.6
54.8
48.5
52.2
48.6
57.1
CD177

Supplementary Figure 11. Intraperitoneal injection of α-TREM-1 for neutrophil transfer. (a–d) IgG or α-TREM-1 was intraperitoneally injected into wild-type mice. Expression of genes related to the TLR signaling pathway (a: *Tlr4*, *Irakm*), autophagy (b: *Atg5*, *Atg7*, *Beclin*), antimicrobial peptides (c: *Reg3b*), and M2 macrophages (d: *Il10*, *Arg1*, *CD206*) was analyzed in colons. (e–j) α-TREM-1-induced $Cd177^+$ neutrophils confer anticolitic effects. Bone marrow-derived neutrophils (BMDNs) were isolated, treated with IgG or α-TREM-1 (0.8 μg/mL) for 24 h, and sorted by FACS to $CD177^+$ and $CD177^-$, which were intraperitoneally injected (1×10^6 cells) into recipient mice 2 days after 3.5% DSS treatment. (e) Flow cytometric analysis of BMDN isolates for neutrophil transfer to mice. (f) Colon length. (g) Gating strategy of flow cytometric analysis of lamina propria mononuclear cells (LPMCs). Data are expressed as means ± S.E.M. (n = 4–5). α-T, treated with α-TREM-1; $CD177^-$ and $CD177^+$, injected with $CD177^-$ or $CD177^+$ neutrophils, respectively; DSS, injected with dextran sulfate sodium. HBSS, injected with HBSS; Veh, treated with vehicle; Water, supplied with normal drinking water. $^*P < 0.05$, $^{**}P < 0.01$, $^{***}P < 0.005$. Analyses were performed using one-way ANOVA with Tukey's posttest.

Supplementary Table

Supplementary Table 1. Clinical and demographic characteristics of healthy controls, Crohn's disease patients, ulcerative colitis patients, and intestinal Behçet's disease patients.

Characteristic	CD	UC	HC
No. of patients	9	11	5
Male/female	8/1	8/3	3/2
Age (yrs, mean ± SD)	31.00 ± 1.31	35.29 ± 12.58	37.80 ± 5.22

CD, Crohn's disease; UC, ulcerative colitis; HC, Healthy control.

Supplementary Table 2. List of sequences of primers used for qRT-PCR

Gene	Sequence (5′–3′)
**Human**	
IL22	F: TGAATAACTAACCCCCTTTCCCTG<br>R: TGGCTTCCCATCTTCCTTTTG
ATG5	F: AAAGATGTGCTTCGAGATGTGT<br>R: CACTTTGTCAGTTACCAACGTCA
IL1B	F: AGCTACGAATCTCCGACCAC<br>R: CGTTATCCCATGTGTCGAAGAA
TNFA	F: ATCTTCTCGAACCCCGAGTG<br>R: GGGTTTGCTACAACATGGGC
TLR4	F: CGGAGGCCATTATGCTATGT<br>R: TCCCTTCCTCCTTTTCCCTA
TREM1	F: CTTGGCAGATAATAAGGGACGG<br>R: CGGACGCGCAGTAAACCAT
BECLIN1	F: ACCTCAGCCGAAGACTGAAG

	R: AACAGCGTTTGTAGTTCTGACA
MUC2	F: AGGATGACACCATCTACCTCACC<br>R: GGTGTAGGCATCGCTCTTCTC
LL37	F: AGGATTGTGACTTCAAGAAGGACG<br>R: GTTTATTTCTCAGAGCCCAGAAGC
KLF4	F: CGGACATCAACGACGTGAG<br>R: GACGCCTTCAGCACGAACT
IL8	F: CTCTTGGCAGCCTTCCTGATT<br>R: TATGCACTGACATCTAAGTTCTTTAGCA

**Mouse**	
Mip2	F: AGTGAACTGCGCTGTCAATG<br>R: CTTTGGTTCCGTTGAGG
Il10	F: GCCACATGCTCCTAGAGCTG<br>R: CAGCTGGTCCTTTGTTTGAAA
Irakm	F: GCCAGAAGAATACATCAGACAGG<br>R: GTCTAAGAAGGACAGGCAGGAGT
Arg1	F: CTCCAAGCCAAAGTCCTTAGAG<br>R: AGGAGCTGTCATTAGGGACATC
Beclin1	F: ATGGAGGGGTCTAAGGCGTC<br>R: TCCTCTCCTGAGTTAGCCTCT
Trem1	F: GCACAACAGGGTCATTCGGAG<br>R: GCACAACAGGGTCATTCGGAG
Cd206	F: CAGGTGTGGGCTCAGGTAGT<br>R: TGTGGTGAGCTGAAAGGTGA
Tlr4	F: ACCAGGAAGCTTGAATCCCT<br>R: TCCAGCCACTGAAGTTCTGA
Atg7	F: GTTCGCCCCCTTTAATAGTGC

	R: TGAACTCCAACGTCAAGCGG
Atg5	F: TGTGCTTCGAGATGTGTGGTT<br>R: GTCAAATAGCTGACTCTTGGCAA
Il22	F: GGCCAGCCTTGC GATAACA<br>R: GCTGATGTGACAGGAGCTGA
Tnfa	F: CAAAGGGAGAGTGGTCAGGT<br>R: ATTGCACCTCAGGGAAGAGT
Il1b	F: GCAACTGTTCCTGAACTCAACT<br>R: ATCTTTTGG GGTCCGTCAACT
IL10rb2	F: ACCTGCTTTCCCCAAAACGAA<br>R: TGAGAGAAGTCGCACTGAGTC
Cd177	F: ATACCAGTGCTGACCCTTCTG<br>R: CCTCGCAGGTTTTCTCACCA
Inos	F: GGCAGCCTGTGAGACCTTTG<br>R: GCA TTG GAA GTG AAG CGT TTC

F: forward primer, **R**: reverse primer

Supplementary Methods

Mouse colitis model

Mice were maintained on a 12:12-h light:dark cycle under specific pathogen-free conditions. Eight-week-old age- and sex-matched mice were randomly divided into each group. Mice were mildly anesthetized by intraperitoneal (i.p.) administration of 2 mg/kg Zoletil (Virbac Laboratories, Carros, France) and 10 mg/kg xylazine (Rompun, Bayeranimalhealth Co., Suwon, South Korea). Colitis was induced by the administration of 100 μL of 5% (w/v) trinitrobenzene sulfonic acid (TNBS) in 50% ethanol with an isotype control (IgG) or α-TREM-1 (4 or 20 μg/mouse as determined from a previous study[1]; MAB1278; R&D Systems, Minneapolis, MN, USA) into the colon of lightly anesthetized mice via a thin round-tip needle equipped with a 1-mL syringe inserted into the colon 4 cm proximal to the anus. Animals were kept in a head-down vertical position for 2 min to distribute the agents within the entire colon and caecum (day 0). For dextran sulfate sodium (DSS)-induced colitis, mice were given with 3% or 3.5% DSS in their drinking water from day 0 for 6 consecutive days, with i.p. injection of either IgG or α-TREM-1. Throughout the experiment, mice were daily monitored for body weight loss, colitis, and overall mortality. Feces were collected for pyrosequencing analysis and were stored at -70°C until analysis. Two days before sacrifice (day 7), drinking water including DSS was changed to untreated drinking. Three days after TNBS administration and 6 or 9 days after DSS administration, mice were euthanized and their spleens and entire colons from the cecum to the anus were quickly retrieved. After sacrifice, disease activity was scored according to the criteria described below. Colon length was measured between the ileocecal junction and the proximal rectum. The colon was opened longitudinally, gently cleared of stool with phosphate buffer saline (PBS), and then cut into several pieces. Distal colon specimens were fixed in 10% buffered

formalin solution (pH 7.4) for at least 1 day, embedded in paraffin, and cross-sectioned at 0.4 μm using standard protocols. The remaining pieces of colon tissue were used for subsequent extraction of lamina propria mononuclear cells (LPMCs) and for mRNA, protein, and metagenome analyses.

Evaluation of disease activity index

Mice were examined daily for body weight, stool consistency, and the presence of gross blood in the stool or at the anus. Mice were scored using a previously validated disease activity index (DAI) that ranged from 0 to 4, based on the following criteria: stool consistency (0, negative; 1 and 2, loose; 3 and 4, diarrhea), gross bleeding (0, absence; 2 and 3, blood tinged; 4, presence), and weight loss (0, none; 1, 1%–5%; 2, 5%–10%; 3, 10%–20%; 4, >20%).[2] DAI values were calculated according to the formula, DAI = (weight loss + stool consistency + gross bleeding)/3. The severity of colitis was evaluated by an independent observer who was blinded to the treatment.

Histological analysis and immunohistochemistry

Paraffin sections were stained with hematoxylin and eosin (H&E) and periodic acid-Schiff (PAS) stains using standard procedures. Histological examination was performed on the distal colon of each animal. Immunohistochemistry was performed using the following antibodies: anti-mouse CD177 (1:500; Novus Biologicals, CO, USA), anti-mouse IL-22 (1:500, eBioscience, San Diego, CA, USA). High-temperature antigen retrieval was performed by immersing the slides in a water bath at 95–98°C in a 10 mM trisodium citrate buffer (pH 6.0) for 45 min. Nonspecific binding

was blocked by incubating sections for 1 h with normal goat serum diluted in PBS. Samples were blocked for endogenous peroxidase activity using 1% H_2O_2. After overnight incubation at 4°C with primary antibodies, slides were washed with PBS and incubated with the appropriate secondary antibodies, as previously described.[3] Images were obtained using a microscope (Olympus BX41; Olympus Optical, Tokyo, Japan). For each animal, the degree of inflammation and goblet cell loss was evaluated on histological sections stained with H&E or PAS stain, as previously described.[3] Three histological parameters were evaluated: severity of inflammation (0–3), extent of injury (0–3), and crypt damage (0–4).

Depletion of microbiota, neutrophils, and macrophages

To deplete bacteria in the gut, a broad-spectrum antibiotic cocktail (vancomycin [10 mg/kg], neomycin sulfate [30 mg/kg], metronidazole [50 mg/kg], and ampicillin [50 μg/kg]) was administered via drinking water for 2 days before DSS treatment.

To deplete neutrophils, an anti-Gr1 antibody (clone RB6-8C5) was injected twice at a dose of 80 mg/mouse. An IgG2b isotype was used as a control (clone LTF-2). All antibodies were from Bio X Cell (West Lebanon, NH, USA). To deplete monocytes, mice were injected i.p. with clodronate liposomes (clodronateliposomes.org, Amsterdam, Netherlands) twice. To assess neutrophil and monocyte depletion, blood was collected from the tail vein immediately before α-TREM-1 treatment on day 0 and cell populations were enumerated by flow cytometry.

Cohousing and microbiota transfer experiments

For cohousing experiments, WT mice were intraperitoneally treated with IgG- and α-TREM-1 6 times weekly and the feces of mice were collected every day, pooled, and weighed for fecal microbiota transplantation (FMT). Stool (0.7 g) was dissolved in 7 mL of PBS and orally administrated to recipient mice for nine days.

Evaluation of microbial load in tissues and microbiota changes by pyrosequencing

Total DNA was isolated from colon tissue and feces using a FastDNA™ SPIN for Soil Kit (MP Biomedicals, Santa) according to the manufacturer's instructions. For pyrosequencing, genomic DNA was amplified using barcoded primers that target the V3–V4 region of the bacterial 16S rRNA gene. Amplification, pyrosequencing, and basic analysis were performed according to the methods described by Chunlab Inc. (Seoul, Korea),[4] using a 454 GS FLX Titanium Sequencing Systems (Roche, Branford, CT, USA) according to the manufacturer's instruction at Chunlab Inc. After chimera check, sequence reads were identified based on 16S rRNA sequence data in the EzTaxon-e database (http://eztaxon-e.ezbiocloud. net). To detect chimera on reads that contain <97% best hit similarity rate, UCHIME and the non-chimeric 16S rRNA database from EzBioCloud were used. We analyzed the number of sequences, diversity richness (operational taxonomic units [OTUs]) and diversity (Shannon index), and estimated OTU richness (abundance-based coverage estimator and Chao1 indices) for alpha diversity analysis. Rank sum test analysis was used to analyze significant differences regarding alpha diversity. Bacterial community abundance and composition were analyzed using the CLcommunity software (Chunlab Inc.).

Lamina propria cell analyses

Lamina propria cells were isolated as previously described[5].

Bone marrow–derived neutrophil and macrophage preparation

Bone marrow was obtained from sacrificed 8- to 15-week-old wild-type or knockout mice by flushing the femurs and tibias with DMEM, using a syringe with a 22-gauge needle. Cells were released from clumps by drawing the suspension through a syringe with a 22-gauge needle and cell suspensions were passed through a 70-µm-pore cell strainer (BD Falcon, San Diego, CA, USA) and centrifuged for 5 min at 1,000 g to remove tissue debris. Red blood cells were lysed with ACK Lysing Buffer (Thermo Fisher Scientific, Waltham, MA, USA) and washed with DMEM. For neutrophil isolation, the remaining cells were resuspended in MACS buffer and isolated using a neutrophil isolation kit (Miltenyi Biotec, Bergisch Gladbach, Germany). Isolated neutrophils were suspended in HBSS, without Ca^{2+} and Mg^{2+}, and kept on ice prior to testing. Neutrophils (2×10^5 per well) were stimulated with IgG, α-TREM-1, or phorbol 12-myristate 13-acetate for 18 h.

For bone marrow-derived macrophage (BMDM) preparation, bone marrow was obtained, and bone marrow cells were isolated as previously described. Cells were plated in DMEM containing 10% FBS, 1% penicillin–streptomycin–L-glutamine (Life Technologies), and 16% L929-conditioned medium; incubated at 37°C in 5% CO_2; and fed on day 4 by replacing the medium with 16% L929 cell-conditioned medium. At day 7, cells were harvested with a cell scraper and replaced with fresh medium in plates required for each experiment. Adherent cells were then stimulated for 24 h with lipopolysaccharides (LPS, 200 ng/mL) in the presence or

absence of α-TREM-1 (800 ng/mL). After stimulation, cells were centrifuged at 1,000 g for 3 min and the cell-free supernatant was collected and stored at -70°C for cytokine determination.

Human blood neutrophil isolation

Human blood was obtained from ulcerative colitis patients, Crohn's disease patients, and healthy subjects. After collecting blood from each subject, the EDTA-coated blood collected tubes were centrifugated for 15 min at 1,500 g. Buffy coats were transferred to new 15-mL conical tubes. Red blood cells were lysed with RBC Lysis Buffer (Biolegend, San Diego, CA, USA). The remaining cells were resuspended in MACS buffer and isolated using a human neutrophil isolation kit (Miltenyi Biotec, Bergisch Gladbach, Germany). Isolated neutrophils were suspended in HBSS, without Ca^{2+} and Mg^{2+}, and kept on ice prior to testing. Supplementary Table 1 summarizes the patient characteristics. The study involved 5 healthy volunteers, 9 patients with Crohn's disease, and 11 patients with ulcerative colitis.

Immunostaining

HT-29 and RAW 264.7 cells were grown on 8-well chamber slides (Nunc, Rochester, NY, USA) and infected with *S. typhimurium* constitutively expressing GFP or exposed to biotinylated LPS (200 ng/mL) and incubated for the specifically mentioned period. Cells were fixed with a 4% paraformaldehyde solution (pH 7.4) and washed with PBS. Animal tissues or cells were blocked in 1% BSA with normal serum in 0.1% Triton X-100, washed, and incubated with primary antibodies or fluorescently conjugated antibodies. Cell nuclei were stained with Hoechst or DAPI

(blue). Immunohistochemistry was performed as previously described.[7] Images were obtained by light microscopy (Olympus BX41) at ×100, ×200, or ×400 magnification.

Flow cytometric analysis

Cell suspensions were prepared in PBS containing 2% FBS. Cells (1×10^6) were blocked with normal mouse serum (Thermo Fisher Scientific) and stained for 30 min at 4°C with appropriate antibodies, including human and mouse anti-Ly6G, without Ly6C cross-reactivity (1:50; 551460; BD Biosciences); anti-IL-22 (1:50, 1H8PWSR; 1:50, 22URTI; eBioscience); anti-F4/80 (1:200, BM8, eBioscience), anti-CD11b (1:50, ICRF4, eBioscience), anti-CD66b (1:50, ICRF4, eBioscience), anti-CD177 (1:50, 1171A; 1:50, MEM-166; BD Biosciences), anti-CD206 (1:50; C068C2; BioLegend), anti-TLR4 (1:100, UT41, eBioscience), and α-TREM-1 (1:100, TR3MBL1, eBioscience). Data were acquired using a FACSVerse flow cytometer (BD Biosciences) and analyzed using FlowJo software (Tree Star, San Carlos, CA, USA).

Extraction of RNA and quantitative RT-PCR

Total RNA was extracted using TRIzol Reagent (Invitrogen, Carlsbad, CA, USA) and 1 μg was reverse transcribed using a SuperScript First-Strand Synthesis Kit (Invitrogen), according to the manufacturer's protocol. Quantitative PCR (qPCR) reactions contained cDNA, Power SYBR Green Master Mix (Applied Biosystems, Foster City, CA, USA), and the appropriate primer pair. All reactions were performed in triplicate. Amplification was performed using a StepOne Plus real-time PCR system (Applied Biosystems) for 45 cycles using the following thermocycling

steps: 95°C for 30 sec, 60–63°C for 30 sec, and 72°C for 40 sec. qPCR primers are listed in Supplementary Table 2. Finally, gene expression levels were calculated using the relative comparative method using the following equation: relative gene expression = $2^{-(\Delta Ct\ sample - \Delta Ct\ control)}$ and results were reported as the fold change compared to the calibrator or $2^{\Delta Ct}$ after normalization of the transcript level to the average of the endogenous control.

Western blotting

Protein was isolated from tissues or cells using Pierce RIPA buffer (Cat# 89900, Thermo Fisher Scientific) mixed with Halt Protease & Phosphatase Inhibitor Cocktail (Thermo Fisher Scientific). Samples were quantified using a bicinchoninic acid assay kit (Pierce, Rockford, IL, USA). Quantified samples were prepared in sodium dodecyl sulfate (SDS) sample buffer, with or without β-mercaptoethanol (Sigma-Aldrich, St Louis, MO, USA). Prepared samples were boiled at 70°C for 10 min and briefly centrifuged. SDS-polyacrylamide gels divided into stacking (5%) and running (10%) components were freshly prepared and 20 to 100 μg of protein samples were loaded. Electrophoresis was performed in Tris-glycine SDS buffer (iNtRON Biotechnology, Seongnam, Korea). Separated proteins were transferred to PVDF membranes, which were blocked with TBST, supplemented with 5% filtered BSA. Primary antibodies (anti-mouse TREM-1, 1:5,000; anti-mouse LC3b, 1:5,000; anti-mouse COX-2, 1:5,000; anti-mouse iNOS, 1:1,000; ABCAM, Cambridge, UK; XBP1, 1:5,000, NOVUS BIO, Littleton, CO, USA; β-actin, 1:2,000, Santa Cruz) were diluted in 5% filtered BSA and incubated overnight at 4℃ with rocking. Membranes were washed and incubated with horseradish peroxidase (HRP)-conjugated secondary antibodies (anti-rabbit, 1:2,000; goat anti-mouse IgG, 1:5,000) diluted in 5% filtered BSA for 3 h.

Chemiluminescence was detected using ECL Supersignal West Pico Plus (Thermo Fisher Scientific) and signals were imaged using a LAS 4000 mini system (Fujifilm, Tokyo, Japan).

Reactive oxygen species measurement

Carboxy-H2DCFDA (Thermo Fisher Scientific) was used, according to the manufacturer's protocol, to study reactive oxygen species (ROS) production. Cells were loaded with the carboxy-H2DCFDA dye by resuspension in HBSS (pH 7.4) with 1 mM Ca^{2+}, 1 mM Mg^{2+}, 1% (v/v) fetal calf serum (FCS), and 2 μM carboxy-H2DCFDA at 10^7 cells/mL. They were then incubated for 30 min at 37°C and washed twice in fresh HBSS prior to stimulation. Fluorescence was immediately analyzed using flow cytometry.

Wound healing assay

HT-29 colon epithelial cells (5×10^5 cells/well) were cultured in 12-well plates under standard culture conditions until reaching 80% confluence. Next, wounds were created using a plastic tip. The plates were marked to facilitate the identification of the same region at later time points. Next, culture media of wounded HT-29 cells were exchanged with conditioned media of HL-60 cells or treated blood neutrophils from ulcerative colitis patients after stimulation with LPS, FLG, or PAM3, and co-stimulation with α-TREM-1. To estimate the degree of wound healing, images were captured every 24 hours and the percentage closure was calculated using cellSens software (Olympus Optical).
